# Effects of aerobic exercise on components of the metabolic syndrome in older adults with type 2 diabetes *mellitus*: systematic review and meta-analysis

**DOI:** 10.17843/rpmesp.2024.412.12751

**Published:** 2024-06-11

**Authors:** Mercedes Miranda-Tueros, Joshua Ramirez-Peña, Miguel Cabanillas-Lazo, José Luis Paz-Ibarra, Isabel Pinedo-Torres

**Affiliations:** 1 Universidad Científica del Sur, Faculty of Health Sciences, Lima, Peru. Universidad Científica del Sur Universidad Científica del Sur Faculty of Health Sciences Lima Peru; 2 Clinical and Health Effectiveness Network, REDECS, Lima, Peru. Clinical and Health Effectiveness Network REDECS Lima Peru; 3 University of Huanuco, Huanuco, Peru. University of Huanuco UUniversity of Huanuco Huánuco Peru; 4 School of Medicine, Universidad Nacional Mayor de San Marcos, Lima, Peru. Universidad Nacional Mayor de San Marcos School of Medicine Universidad Nacional Mayor de San Marcos Lima Peru; 5 Edgardo Rebagliati Martins National Hospital, Lima, Peru. Edgardo Rebagliati Martins National Hospital Lima Perú; 6 NEURONECS Research Group: Neuroscience, clinical effectiveness and public health, Universidad Científica del Sur, Lima, Peru. Universidad Científica del Sur NEURONECS Research Group: Neuroscience, clinical effectiveness and public health Universidad Científica del Sur Lima Peru

**Keywords:** Exercise, Diabetes Mellitus, Aged, Systematic Review, Metabolic Syndrome

## Abstract

**Objective.:**

To determine the effects of aerobic exercise on the components of the metabolic syndrome in older adult diabetic patients by means of a systematic review with meta-analysis.

**Materials and methods.:**

We used the PubMed/Medline, Scopus, Cochrane library, Web of Science databases and the Google Scholar search engine. Randomized controlled trials (RCTs) were selected according to the inclusion criteria. Two reviewers independently determined whether studies met the inclusion criteria, extracted data, and used the Cochrane risk of bias tool (RoB 2). Quantitative analyses were performed in R v 4.0.5, using random effects.

**Results.:**

We identified 8697 studies, of which 7 RCTs were included in the qualitative synthesis. Most studies were assessed as having a high or low RoB in at least three domains. Meta-analysis showed that aerobic exercise was effective in improving glucose levels (standardized mean difference [SMD]: -1.04; 95% confidence interval [95% CI] -1.27, -0.81), systolic blood pressure (SMD: -0.79; 95% CI: -1.02, -0.56), diastolic blood pressure (SMD: -0.75; 95% CI: -0.98, -0.52), glycosylated hemoglobin (SMD: -0.57, 95% CI: -0.77, -0.37), HDL (SMD: 0.35, 95% CI: 0.15, 0.55), triglycerides (SMD: -0.26, 95% CI: -0.47, -0.06). No significant adverse effects were reported. The level of certainty of the results was low for fasting glucose, moderate for systolic and diastolic blood pressure, and very low for the other outcomes, in addition to few adverse effects. However, these results should be interpreted with caution due to the use of surrogate markers.

**Conclusions.:**

Aerobic exercise was shown to have a significant improvement in the components of the metabolic syndrome in older diabetic adults, and no major adverse effects were reported. However, we recommend more RCTs with longer intervention time to establish the impact on symptoms and complications.

## INTRODUCTION

It is estimated that by the year 2050, the population of older adults will double the projection reported for 2019, which means that one in six people in the world will be 65 years of age or older ^1^. In addition, metabolic syndrome, and its complication, diabetes mellitus, is very frequent in this population ^2^^,^^3^. This syndrome includes the alteration of three or more of the following signs: systolic blood pressure, diastolic blood pressure, high-density lipoprotein cholesterol, triglycerides, and fasting glucose; the assessment of glycosylated hemoglobin is more useful in people with diabetes due to its high clinical value and good diagnostic accuracy in complications of hyperglycemia ^4^. It has even been used in conjunction with fasting glucose as a screening method for metabolic syndrome and it has been considered as a possible criterion for metabolic syndrome ^5^^,^^6^. It should be noted that the presence of these two conditions (diabetes and metabolic syndrome) increases the risk of fatal cardiovascular complications in the short term, such as cardiac ischemia or cerebrovascular disease ^7^^,^^8^.

Currently, available guidelines recommend physical activity along with diet and medication to improve glycemic and lipid parameters in patients with diabetes ^9^^,^^10^^).^ There are several types of proven useful exercises when it comes to physical training, such as aerobic, resistance and high intensity exercise ^11^^,^^12^. Aerobic exercise, defined as any work that develops cardiovascular and pulmonary fitness, uses oxygen as the metabolic substrate ^13^ and is the most studied. This includes workouts of varying intensities, from brisk walking to jogging or swimming ^14^^,^^15^. This type of exercise provides several benefits, such as improved insulin sensitivity, increased fibrinolysis, lower triglycerides and blood pressure, all of which improve the prognosis of a patient with diabetes ^16^^-^^19^.

However, physical exercise cannot be prescribed without restrictions. The older adult population often has limited mobility, obesity, visual impairment or cardiovascular disease, and the geriatric population diagnosed with diabetes is at increased risk of hypoglycemia and frailty syndrome ^20^^-^^23^. Although guidelines ^24^ recommend that older adults perform at least 150 minutes of moderate-intensity aerobic physical activity or 75 minutes of vigorous-intensity aerobic physical activity per week, the potential effects of aerobic exercise on metabolic markers for the adequate control of diabetes in older people and the safety of its prescription are not fully understood ^25^. Therefore, this systematic review with meta-analysis aimed to determine the effects of aerobic exercise on the components of the metabolic syndrome in older adult patients with diabetes in order to provide general practitioners and specialists with a comprehensive overview of the scientific evidence on a possible non-pharmacological treatment option to improve the health of this population.

**KEY MESSAGES**

Motivation for the study. The motivation for this research arises from the high prevalence of metabolic syndrome and diabetes mellitus around the world. Despite their impact, there is a gap in knowledge regarding non-pharmacological interventions in older adults aimed at improving the metabolic profile of these patients.

Main findings. Our results show a significant improvement in glucose, blood pressure, glycosylated hemoglobin, HDL, and triglyceride levels after the aerobic exercise intervention. In addition, no significant adverse effects were observed.

Public health implications. Physical exercise is an affordable and globally available strategy. It improves the metabolic profile of older adult patients with metabolic syndrome.

## MATERIALS AND METHODS

A systematic review was carried out following the guidelines of the “Systematic Reviews and Meta-Analyses (PRISMA) 2020”. The protocol was registered in PROSPERO with the code CRD42021250115.

### Search strategy

A systematic search was carried out in PubMed/Medline, Scopus, Cochrane library, Web of Science databases and the Google Scholar search engine was used for gray literature. MeSH and free related terms were used for “exercise”, “diabetes mellitus”. Instead of including truncators, related terms such as “jogging”, “treadmill”, “swimming”, “running”, or “ambulation” were expanded to the main terms (supplementary material).

### Selection criteria

We included randomized clinical trials (RCTs) that met the following criteria: a) patients with type 2 diabetes *mellitus* (DM2) with at least one of the components of the metabolic syndrome, b) evaluation of older adults (over 60 years of age), c) availability of full text of the article and d) intervention of any type of aerobic exercise for a minimum time of 12 weeks compared to standard of care or no intervention. We used the definition of aerobic exercise from the “Physical Activity Guidelines for Americans” by the U.S. Department of Health and Human Services, which states that aerobic exercise is the movement of large muscles of the body in a rhythmic manner for a sustained period of time ^14^. Aerobic exercise includes running, walking, ergometer cycling and treadmill use ^27^. We classified exercise intensity as sedentary (<1.6 METs, <40% HRmax, <20% HRR, <20% VO_2_ max, RPE (C): <8, RPE (C-R): <1), mild (1.6-3 METs, 40-55% HRmax, 20-40% HRR, 20-40% VO2 max, RPE (C): 8-10, RPE (C-R): 1-2), moderate (3-6 METs, 55-70% HRmax, 40-60% HRR, 40-60% VO2 max, RPE (C): 11-13, RPE (C-R): 3-4) and vigorous (6-9 METs, 70-90% HRmax, 60-85% HRR, 60-85% VO_2_ max, RPE (C): 14-16, RPE (C-R): 5-6). These intensity measures are abbreviated with the following terminology, MET stands for “metabolic equivalent”, where 1 MET equals 3.5 ml O_2_/kg/min; % HRmax is the “maximum heart rate”; %HRR alludes to the “heart rate reserve”, which is the maximum HR - resting HR; and %VO_2_ max is the “maximum oxygen consumption”. On the other hand, we used subjective measures from the Borg’s RPE scales of perceived exertion, where RPE-C, measures on a category ratio scale of 6-20 and RPE C-R measures on the category ratio scale of 0-10 ^27^. Search was not restricted by year. Other types of publication (letters to the editor, case reports, observational studies, narrative and systematic reviews) were excluded.

### Study selection and data extraction

Search results were imported into the EndNote X9 reference management program, then duplicate records were removed, according to the procedures described by Bramer *et al*. ^26^. Subsequently, two authors (MMT and JRP) independently screened the titles and abstracts according to the selection criteria. We selected relevant studies and searched for the full-text articles. Discrepancies in the selections were solved by consensus and, ultimately, a third author (IPT) was consulted. The full list of full-text articles excluded at this stage is available in Supplementary Material 2. Two authors (JRP and MCL) independently extracted data for each included article using a standardized form in Microsoft Excel. A third author (IPT) verified the accuracy of the data prior to analysis. Absolute and relative frequencies were obtained for dichotomous outcomes. Baseline, follow-up measurements and the change between them were obtained for continuous outcomes.

### Analysis of results

The primary outcomes were the components of the metabolic syndrome according to the International Diabetes Federation (IDF) ^27^: fasting glucose (mg/dl), systolic blood pressure (SBP, mmHg), diastolic blood pressure (DBP, mmHg), high-density lipoprotein (HDL, mg/dl), triglycerides (mg/dl) and waist circumference (cm/inch). Glycosylated hemoglobin (HbA1c, %) was also considered due to its clinical importance, despite not being part of the components of the metabolic syndrome. Cohen’s rules were used to interpret the effect size ^28^.

### Analysis by subgroups

Three key variables were considered for this analysis: exercise intensity, exercise monitoring and total duration in hours. Three intensity categories were established: high or vigorous (≥ 6 METs, maximum heart rate ≥ 70%), low or moderate (< 6 METs, maximum heart rate < 70%) ^14^ and not reported. On the other hand, we evaluated whether the exercise, according to the trial, was supervised by any health professional, personal trainer in a face-to-face or virtual manner. We also considered the total duration of the exercise in hours, which was categorized in two: greater than 30 hours and less than or equal to 30 hours per month, according to a study by Shiroma *et al*., who determined that those patients who performed less than 60 minutes had an increase in weight greater than or equal to 3% in the following five years in older adults ^29^.

### Bias risk assessment

Randomized clinical trials were assessed using the Cochrane risk of bias tool for randomized trials (RoB 2) ^30^. This tool considers several domains in which bias could have arisen, such as the randomization process, deviations from planned interventions (intervention allocation effect), missing outcome data, outcome measurement, and selection of reported outcomes. A judgment-based algorithm was used for each domain in order to determine whether there was low risk, some concern, or high risk of bias. Randomized clinical trials were considered to have high risk of bias if they were at high risk in any of the assessed domains. The RoB 2 assessment was performed independently by two authors (MCL, MMT), and discrepancies were solved by discussion or consultation with a third author (IPT).

### Statistical analysis

The inverse variance method and the random-effects model were used for all meta-analyses. The between-study variance (τ2) was estimated using the DerSimonian-Laird tool. The pooled effect of each outcome was determined by the standardized mean difference (SMD) of the change values between intervention and control groups with 95% confidence intervals (95% CI). The Cochrane Handbook method was used through imputation of correlation coefficients when the standard deviation of mean differences was not reported ^31^. Heterogeneity between studies was assessed using the I2 statistic. Heterogeneity was defined as low if I2 < 30%, moderate if I2 = 30-60%, and high if I2 > 60%. All of the above was presented as a forest plot graph for each outcome.

Each subgroup was analyzed separately in order to assess the source of heterogeneity, these analyzes were performed according to exercise intensity, exercise performance monitoring and total intervention time. The *metacont* function of the R 4.3.0 statistical package (www.r-project.org) was used.

### Certainty of evidence

The Grading of Recommendations Assessment, Development and Evaluation (GRADE) approach was used to assess the quality of evidence for all outcomes ^32^. When applying the GRADE methodology, the following criteria were taken into account to assess the quality of the studies: risk of bias, inconsistency, imprecision (a difference of one SD was taken into account as the minimum difference), publication bias, large magnitude of effect, dose-response gradient, plausible residual confounding effect, and level of indirectness. The summary of findings (SOF) table was generated using GRADE pro software (https://www.gradepro.org). The assessment of certainty of evidence using this scale was performed by the authors MMT and MCL, in duplicate and following GRADE recommendations.

### Ethical aspects

All clinical trials included in the study had the necessary ethical approval. The extracted information underwent quality control at each stage to ensure the fidelity of the data. In addition, a protocol was prepared before the start of the research, which is publicly available in PROSPERO (https://www.crd.york.ac.uk/prospero/) with the registration code CRD42021250115 and guarantees the transparency of the process.

## RESULTS

**Figure 1:**
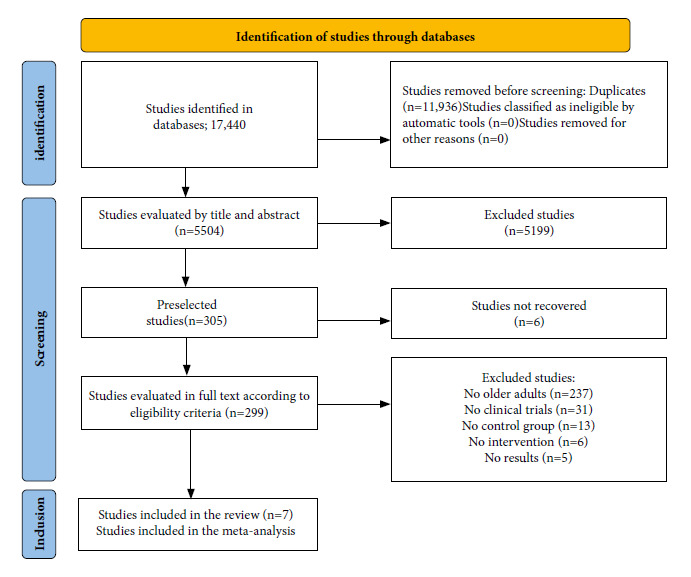
PRISMA study selection diagram

A total of 17,440 abstracts were identified from the databases and 11,936 duplicates were eliminated. After evaluation by title and abstract, 299 were left for full-text evaluation and six were not accessed. Finally, seven articles met the inclusion criteria. The selection process is described in Figure 1.

### Characteristics of the included studies

The main characteristics of the seven studies are summarized in Supplementary Material 3. A total of 386 participants with a mean age ranging from 62.9 to 73.2 years were evaluated. Two studies were from Canada. Outdoor or treadmill walking was the intervention in all of the studies ^33^^-^^39^. The duration of the intervention ranged from 12 to 24 weeks and, among those reporting exercise intensity, 50% were of high intensity. Of the seven included trials, one showed a high risk of bias overall, as noted in Supplementary Material 4.

### Primary outcomes

**Figure 2:**
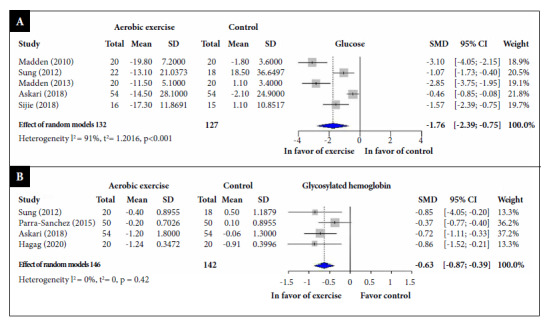
Standardized mean difference between aerobic exercise and control groups, a random effects model meta-analysis. (A) Fasting blood glucose; (B) Glycosylated hemoglobin

Meta-analyses were conducted for each outcome with all seven included trials. The results are detailed below: 

In relation to fasting glucose, in five RCTs ^34^^,^^35^^,^^40^^-^^44^ involving 259 participants, aerobic exercise significantly reduced glucose values (SMD: -1.76; 95% CI: -2.78, -0.74; I2 = 91%) compared to the control group (Figure 2A). 

Regarding glycosylated hemoglobin, four RCTs ^34^^,^^37^^,^^45^^-^^49^ involving 288 participants, reported that aerobic exercise significantly reduced HbA1c values (SMD: -0.63; 95% CI: -0.87, -0.39; I2 = 0%) compared to the control group (Figure 2B).

**Figure 3:**
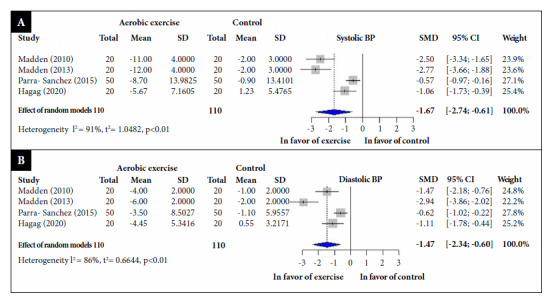
Standardized mean difference between aerobic exercise and control groups, a random effects model meta-analysis. (A) Systolic blood pressure; (B) Diastolic blood pressure.

With respect to systolic blood pressure, four RCTs, involving 220 participants ^33^^,^^34^^,^^36^^,^^39^^,^^48^^,^^50^^)^ reported that aerobic exercise significantly reduced SBP values (SMD: -1.67; 95% CI: -2.74, -0.61; I2 = 90%) compared to the control group (Figure 3A).

Regarding diastolic blood pressure, four RCTs ^33^^,^^34^^,^^36^^,^^39^^,^^47^^,^^50^^)^ involving 220 participants, reported that aerobic exercise significantly reduced DBP values (SMD: -1.47; 95% CI: -2.34, -0.60; I2 = 86%) compared to the control group (Figure 3B).

**Figure 4:**
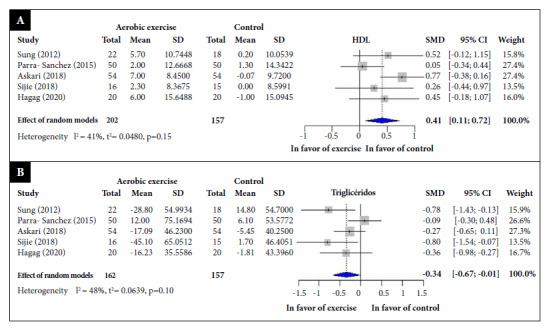
Standardized mean difference between aerobic exercise and control groups, a random effects model meta-analysis. (A) High-density lipoprotein; (B). Triglycerides

Regarding HDL levels, five RCTs ^35^^-^^39^^,^^47^, which included 319 participants, reported that aerobic exercise significantly increased their values (SMD: 0.41; 95% CI: 0.11, 0.72; I2 = 41%) compared to the control group (Figure 4A).

Regarding triglyceride levels, five RCTs ^35^^-^^39^^,^^48^ involving 319 participants, reported that aerobic exercise significantly reduced triglyceride values (SMD: -0.34; 95% CI: -0.67, -0.01; I2 = 48%) compared to the control group (Figure 4B).

A meta-analysis of waist circumference was not performed since we only found one RCT ^38^; however, there was a significant reduction between the aerobic exercise group and the control group (p < 0.001).

Regarding sensitivity analysis, no individual study significantly affected the aggregate result in any outcome (see supplementary material 5).

### Adverse effects

Two of the seven trials reported some adverse effects (supplementary material 3). Madden *et al*. ^34^ described one case of carotid massage-related syncope that occurred both during and after exercise. For their part, Parra-Sanchez *et al*. ^36^ reported one case of sprain in the intervention group, and two cases of stroke, one in the intervention group and the other in the control group. Adverse effects were not considered as additional outcomes due to their scarcity in the included studies.

### Analysis by subgroups

A subgroup analysis was performed to evaluate the results based on some variables such as exercise intensity, exercise performance monitoring and total intervention time (supplementary material 6). 

Regarding fasting glucose levels and systolic and diastolic blood pressure, all subgroups were statistically significant, except for most trials that did not report exercise intensity.

Regarding decreasing triglycerides, trials that did not report exercise intensity, those with a total duration of intervention less than or equal to 30 hours, and those with or without monitoring of exercise compliance had no significant differences between the intervention and control group.

Then, regarding HDL elevation, only the trials that did not monitor exercise performance showed a statistically significant difference between the intervention group and the control group. Finally, regarding the decrease in HbA1c, all subgroups were significant.

### Certainty of evidence

The certainty of evidence was evaluated at four levels, from high to very low, according to the degree of confidence in the estimate of the effect. Results show that the certainty of evidence is low for the decrease of fasting glucose and low for SBP and DBP. This is due to the high heterogeneity between studies and the difference in the type of aerobic exercise. The certainty of evidence for high-density lipoprotein-associated cholesterol, triglycerides, and HbA1c is very low. This is because the confidence intervals for these effects are imprecise; there are concerns in different domains of risk of bias assessment, as well as difference between the types of aerobic exercise meta-analyzed (Table 1).

**Table 1:**
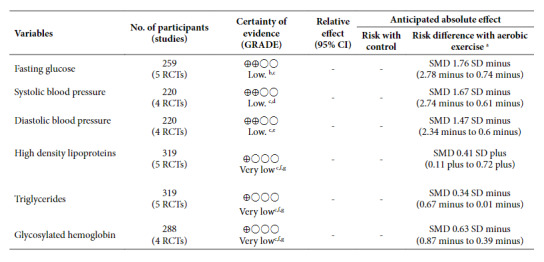
Certainty of evidence (GRADE).

CI, confidence interval; SD, standard deviation; SMD, standardized mean difference.

a The risk in the intervention group (and its 95% confidence interval) are based on the risk assumed in the control group and the relative effect of the intervention (and its 95% confidence interval), ^b^ the value of I^2^ is 91% in this variable, ^c^ different type and duration of interventions, ^d^ the value of I2 is 90% on this variable, ^e^ the value of I2 is 86% on this variable, ^f^ imprecise confidence intervals, ^g^ most of the studies have “some concerns” in domain one.

GRADE Evidence Certainty Groups. High certainty: we are very confident that the true effect is close to the effect estimate. Moderate certainty: we have moderate confidence in the effect estimate: the true effect is likely to be close to the effect estimate, but there is a possibility that it is substantially different. Low certainty: our confidence in the estimate of effect is limited: the true effect may be substantially different from the estimate of effect. Very low certainty: we have very low confidence in the estimate of effect: the true effect is likely to be substantially different from the estimate of effect.

## DISCUSSION

This systematic review provides a comprehensive and up-to-date overview of the effects of aerobic exercise on the components of the metabolic syndrome in the geriatric population. We found a significant decrease in fasting glucose, glycosylated hemoglobin, triglycerides, SBP, DBP, and increased HDL values. In addition, four of the 386 study participants had adverse events and in one participant in the intervention group, the event was severe (stroke). Additionally, all primary outcomes assessed had either a low or very low level of certainty.

From the synthesis of five RCTs, we found that glucose values decreased with the practice of aerobic exercise. Our findings were similar to those reported by Kumar *et al*. ^44^ who evaluated 11 RCTs with an adult population of diabetic patients and found a decrease in both fasting glucose and HbA1c. These results may be explained by the fact that a frequent training routine improves hepatic performance by reducing lipid accumulation, improving insulin resistance in this organ and decreasing nocturnal hepatic glucose production ^45^, along with increased mitochondrial enzyme activity (thus improving muscle energy) ^46^. 

The results of the synthesis of four RCTs in our review, as well as other systematic reviews with similar characteristics, are consistent when HbA1c is used as a measure of glycemic control. These results are similar to what has been found in other systematic reviews with similar characteristics. For example, in a similar population, Pan *et al*. ^38^^)^ demonstrated that supervised aerobic exercise had a greater effect on HbA1c compared with resistance or combined exercise. In addition, Hagag *et al*. ^39^^)^ found a mean difference of -0.46 in HbA1c in favor of aerobic exercise in a sample of 972 participants. It is believed that the difference in the substrate used during each type of exercise could explain these results. More glucose is used during high intensity exercise, on the other hand, more fatty acids are used during moderate exercise ^41^.

Regarding lipid metabolism, our review synthesized the results of five RCTs that support the relationship between aerobic exercise and normalization of lipid and lipoprotein values. Particularly, an increase in HDL and a decrease in triglycerides were found after the intervention. These results are consistent with other reviews such as that of Schwingshacklet *et al*. ^47^, who reported improvements in all components of the lipid profile after the practice of different types of exercise for eight weeks, especially with combined exercise. The increase in HDL with exercise is due to the association between aerobic exercise and increased lipoprotein lipase activity, which results in increased lipolysis of triglyceride-rich lipoproteins, which may be an initial step in higher serum HDL levels. In addition to the above, there is a reduction in hepatic lipase activity and serum cholesteryl ester transfer protein (CETP) concentration, the latter being the one that catalyzes the transformation flux from HDL cholesterol to VLDL cholesterol and LDL cholesterol ^48^. 

The synthesis of four RCTs showed that aerobic exercise decreases SBP and DBP values. In this regard, Punia *et al*. ^49^ found that aerobic exercise had a significant effect on decreasing SBP and DBP in Indian adults. The mechanism by which this occurs is a reduction in peripheral vascular resistance leading to a decrease in systolic and diastolic blood pressure values after training ^17^. However, according to another study, the mechanisms by which exercise reduces blood pressure are not yet fully understood. Several possible pathways have been proposed, including reduced inflammation, decreased oxidative damage, control of sodium sensitivity, and decreased arterial stiffness ^46^.

Regarding adverse effects, one serious adverse event (stroke) was found in one of the 386 participants included in the study. In another RCT that evaluated different training routines in 221 individuals, Church *et al*. ^50^^)^ reported five cardiovascular events, but none related to physical exercise. However, it should be noted that previous studies have reported greater adverse effects in older adults with diabetes, possibly due to their frailty and higher prevalence of comorbidities ^50^. Therefore, the results of the trials should be viewed with caution, particularly considering that the mean age of the participants in the trials ranged between 60 and 70 years. 

Considering that diabetes is one of the major public health issues ^10^, our study shows that aerobic exercise, a low-cost and relatively easy to implement measure, greatly helps to improve the components of the metabolic syndrome in the elderly population. Its implementation in the healthcare system as a dedicated aerobic activity program could potentially reduce the occurrence of complications significantly and prevent short-term progression to cardiovascular events, which have a high mortality and a high risk of irreversible sequelae and dependency. In addition, indirect beneficial effects can be added that greatly reduce frailty in this age group. However, because the analyzed studies are based on surrogate outcomes, clinical relevance cannot be confirmed. Last but not least, it would probably help to reduce the dose of hypoglycemic and hypotensive drugs, which could translate into lower cost in chronic disease maintenance. Our review is relevant because, to our knowledge, it is the first to analyze the effects of aerobic exercise on the components of the metabolic syndrome in the aforementioned population. This would help to guide future management and implement this type of exercise in the daily practice of these patients.

Our research had some limitations. One of the main limitations is that we found a high percentage of studies with high or moderate risk of bias. Another limitation is that the characteristics of the interventions were heterogeneous, however, we performed a subgroup analysis according to intervention time, supervision and intensity, in which most showed no differences; these results should be viewed with caution due to the low number of trials. Then, this study covered seven RCTs, the vast majority of these were from Asian countries and had relatively low numbers of participants, although a sensitive analysis was performed excluding each study, which showed no significant differences. However, more studies of European and American populations are needed. Another one of the limitations was the heterogeneity of the studies included in the meta-analysis such as fasting blood glucose and systolic and diastolic blood pressure. 

Finally, it was not possible to evaluate the specific effect of different aerobic exercise routines because the intervention was the same or similar (treadmill, walking, treadmill and ergometer bicycle) in most of the included RCTs. Nevertheless, all these types of exercise stimulate large portions of skeletal muscle, thereby causing favorable changes in the cardiovascular system ^24^.

In conclusion, the available clinical studies show a significant decrease in fasting glucose, triglycerides, glycosylated hemoglobin, SBP and DBP values, as well as a small modification of triglycerides and HDL in older adults with DM2 who performed aerobic exercise compared to controls without exercise, so we suggest to apply this intervention in this population considering the level of certainty of evidence being low for fasting glucose, systolic and diastolic blood pressure, and very low for the other outcomes. The results of the study were also very positive, in addition to few adverse effects. However, these results should be interpreted with caution due to the use of surrogate markers. Finally, clinical trials of better methodological quality, multicenter and with larger samples are recommended to provide a correct representation of the effects of aerobic exercise.
