# Supplementary material for: Effects of aerobic exercise on components of the metabolic syndrome in older adults with type 2 diabetes mellitus: systematic review and meta-analysis
Source: Rev Peru Med Exp Salud Publica. 2024 Jun 11;41(2):146–55. doi: 10.17843/rpmesp.2024.412.12751 (PMC11300696; doi:10.17843/rpmesp.2024.412.12751)
Supplement: Supplementary material. — Available in the electronic version of the RPMESP. [file rpmesp-41-02-12751-s001.docx]

**MATERIAL SUPLEMENTARIO**

**Material suplementario 1**. Búsqueda electronica

| **Bases de datos** | **Estrategia de búsqueda** | **Numero de resultados** |
| --- | --- | --- |
| **Pubmed/**  **Medline** | #1 (“Diabetes Mellitus”[MeSH] OR “Diabetes Mellitus Type 2”[tiab] OR “Diabetes Mellitus Noninsulin-Dependent”[tiab] OR “Diabetes Mellitus Non Insulin Dependent”[tiab] OR “Diabetes Mellitus Non-Insulin-Dependent”[tiab] OR “Non-Insulin-Dependent Diabetes Mellitus”[tiab] OR “Diabetes Mellitus Stable”[tiab] OR “Diabetes Mellitus Type II”[tiab] OR “NIDDM”[tiab] OR “Diabetes Mellitus Noninsulin Dependent”[tiab] OR “Diabetes Mellitus Slow-Onset”[tiab] OR “Type 2 Diabetes”[tiab])  #2 (“exercise”[MeSH] OR “Exercises”[tiab] OR “Physical Activity”[tiab] OR “Activities, physical”[tiab] OR “activity, physical”[tiab] OR “physical activities”[tiab] OR “Exercise Physical”[tiab] OR “Exercises, physical”[tiab] OR “Physical Exercise”[tiab] OR “physical exercises”[tiab] OR “Aerobic Exercise”[tiab] OR “Aerobic Exercises”[tiab] OR “Exercises Aerobic”[tiab] OR “Exercise Training”[tiab] OR “Exercise Trainings”[tiab] OR “Training Exercise”[tiab] OR “Aerobic training”[tiab] OR "endurance training"[MeSH] OR “Training Endurance”[tiab] OR “Physical endurance”[tiab] OR "sports"[MeSH] OR “Sport”[tiab] OR “Athletic*”[tiab] OR "walking"[MeSH] OR “Ambulation”[tiab] OR "running"[MeSH] OR “Runnings”[tiab] OR “Swimming”[tiab] OR “Treadmill”[tiab] OR “Treadmill Test”[tiab] OR “Treadmill Tests”[tiab] OR “Jogging”[MeSH])  #3 ("Randomized Controlled Trial"[PT] or "Randomized Controlled Trials as Topic"[Mesh] OR "Clinical Trial"[PT] OR "Clinical Trials as Topic"[Mesh] OR "Clinical Trial, Phase III"[PT] OR "Clinical Trial, Phase II"[PT] OR "Double-Blind Method"[Mesh] OR "Random Allocation"[Mesh] OR "Single-Blind Method"[Mesh] OR (random*[tiab]) OR ((randomized[tiab] OR randomised[tiab] OR clinical[tiab] OR control*[tiab]) AND trial[tiab]) OR ((singl*[tiab] OR doubl*[tiab] OR trebl*[tiab] OR tripl*[tiab]) AND (blind*[tiab] OR mask*[tiab])) OR (“Placebos”[Mesh] OR placebo*[tiab]) OR ("Cross-Over Studies"[Mesh]) OR ((crossover[tiab] OR cross-over[tiab] OR “cross over”[tiab]) AND (design*[tiab] OR study[tiab] OR studies[tiab] OR procedure*[tiab] OR trial*[tiab])))  #4 #1 AND #2 AND #3 | 3787 |
| **Scopus** | #1 (TITLE-ABS-KEY("Exercise" OR "Exercises" OR "Aerobic training" OR "Physical Activity" OR "Physical Activities" OR "Physical Exercise" OR "Physical Exercises" OR "Aerobic Exercise" OR "Aerobic Exercises" OR "Exercise Training" OR "Exercise Trainings")) OR (TITLE-ABS-KEY("Endurance training" OR "Physical endurance")) OR (TITLE-ABS-KEY("Treadmill" OR "Treadmill Test" OR "Treadmill Tests")) OR (TITLE-ABS-KEY("Pools Swimming" OR "Swimming Pool")) OR (TITLE-ABS-KEY("Jogging")) OR (TITLE-ABS-KEY ("Sports" OR "Sport" OR "Athletics" OR "Athletic")) OR (TITLE-ABS-KEY("Walking" OR "Ambulation")) OR (TITLE-ABS-KEY("Running" OR "Runnings")) OR (TITLE-ABS-KEY("Jogging"))  #2: TITLE-ABS-KEY ("Diabetes Mellitus Non Insulin Dependent" OR "Diabetes Mellitus Noninsulin Dependent" OR "Non Insulin Dependent Diabetes Mellitus" OR "Noninsulin Dependent Diabetes Mellitus" OR "Diabetes Mellitus Stable" OR "Stable Diabetes Mellitus" OR "NIDDM" OR "Diabetes Mellitus Slow Onset" OR "Slow Onset Diabetes Mellitus" OR "Type 2 Diabetes Mellitus" OR "Type 2 Diabetes" OR "Diabetes Mellitus Type 2" OR "Diabetes Type 2" OR "Diabetes Mellitus Type II" OR "Diabetes Type II" OR "Type II Diabetes Mellitus" OR "Type II Diabetes" OR "Diabetes mellitus 2" OR "Diabetes mellitus II")  #3: ((TITLE-ABS-KEY ("randomized" OR "randomised" OR "clinical" OR "control*") AND TITLE-ABS-KEY ("trial")) OR (TITLE-ABS-KEY("singl*" OR "doubl*" OR "trebl*" OR "tripl*") AND TITLE-ABS-KEY("blind*" OR "mask*")) OR (TITLE-ABS-KEY("crossover" OR "cross-over" OR "cross over") AND TITLE-ABS-KEY("design*" OR "study" OR "studies" OR "procedure*" OR "trial*")) OR TITLE-ABS-KEY("Randomized Controlled Trial" OR "Randomized Controlled Trials as Topic" OR "Clinical Trial" OR "Clinical Trials as Topic" OR "Clinical Trial, Phase III" OR "Clinical Trial, Phase II" OR "Double-Blind Method" OR "Random Allocation" OR "Single-Blind Method" OR "random*" OR "placebo*"))  #4 ( LIMIT-TO ( PUBYEAR,2021) OR LIMIT-TO ( PUBYEAR,2020) OR LIMIT-TO ( PUBYEAR,2019) OR LIMIT-TO ( PUBYEAR,2018) OR LIMIT-TO ( PUBYEAR,2017) OR LIMIT-TO ( PUBYEAR,2016) OR LIMIT-TO ( PUBYEAR,2015) OR LIMIT-TO ( PUBYEAR,2014) OR LIMIT-TO ( PUBYEAR,2013) OR LIMIT-TO ( PUBYEAR,2012) OR LIMIT-TO ( PUBYEAR,2011) OR LIMIT-TO ( PUBYEAR,2010) )  #5 ( LIMIT-TO ( DOCTYPE,"ar" ) )  #6: #1 AND #2 AND #3 AND #4 AND 5 | 5044 |
| **Web of science** | #1: TS=("Exercise" OR "Exercises" OR "Aerobic training" OR "Physical Exercise" OR "Physical Exercises" OR "Aerobic Exercise" OR "Aerobic Exercises" OR "Exercise Training" OR "Exercise Trainings" OR "Endurance training" OR "Physical endurance" OR "Treadmill" OR "Treadmill Test" OR "Treadmill Tests" OR "Pools Swimming" OR "Swimming Pool" OR "Jogging" OR "Sports" OR "Sport" OR "Athletics" OR "Athletic" OR "Walking" OR "Ambulation" OR "Running" OR "Runnings")  #2: TS=("Diabetes Mellitus Non Insulin Dependent" OR "Diabetes Mellitus Noninsulin Dependent" OR "Non Insulin Dependent Diabetes Mellitus" OR "Noninsulin Dependent Diabetes Mellitus" OR "Diabetes Mellitus Stable" OR "Stable Diabetes Mellitus" OR "NIDDM" OR "Diabetes Mellitus Slow Onset" OR "Slow Onset Diabetes Mellitus" OR "Type 2 Diabetes Mellitus" OR "Type 2 Diabetes" OR "Diabetes Mellitus Type 2" OR "Diabetes Type 2" OR "Diabetes Mellitus Type II" OR "Diabetes Type II" OR "Type II Diabetes Mellitus" OR "Type II Diabetes" OR "Diabetes mellitus 2" OR "Diabetes mellitus II")  #3: TS=(randomized OR randomised OR clinical OR control*) AND TS=(trial)  #4: TS=(singl* OR doubl* OR trebl* OR tripl*) AND TS=(blind* OR mask*)  #5: TS=(crossover OR cross-over OR "cross over") AND TS=(design* OR study OR studies OR procedure* OR trial*)  #6: TS=("Randomized Controlled Trial" OR "Randomized Controlled Trials as Topic" OR "Clinical Trial" OR "Clinical Trials as Topic" OR "Clinical Trial, Phase III" OR "Clinical Trial, Phase II" OR "Double-Blind Method" OR "Random Allocation" OR "Single-Blind Method" OR random* OR placebo*)  #7: #3 OR #4 OR #5 OR #6  #8: #1 AND #2 AND #7 | 3787 |
| **Cochrane** | #1 MeSH descriptor: [Exercise] explode all trees  #2 (“Exercises“ OR “Physical Activity” OR “Activities Physical” OR “Activity Physical” OR “Physical Activities” OR “Exercise Physical” OR “Exercises Physical” OR “Physical Exercise” OR “Physical Exercises” OR “Exercise Aerobic” OR “Aerobic Exercise” OR “Aerobic Exercises” OR “Exercises Aerobic” OR “Exercise Training” OR “Exercise Trainings” OR “Training Exercise” OR “Trainings Exercise” OR “Aerobic training”):ti,ab,kw  #3 MeSH descriptor: [Endurance Training] explode all trees  #4 (“Training Endurance” OR “Phyisical endurance”):ti,ab,kw  #5  MeSH descriptor: [Sports] explode all trees  #6 (sport OR athletics OR athletic):ti,ab,kw  #7 MeSH descriptor: [Swimming] explode all trees  #8 (swimming):ti,ab,kw  #9 MeSH descriptor: [Walking] explode all trees  #10 (ambulation):ti,ab,kw  #11 MeSH descriptor: [Running] explode all trees  #12 (runnings):ti,ab,kw  #13 ("treadmill" OR "treadmill test" OR "test, treadmill" OR "treadmill tests" OR "jogging"):ti,ab,kw  #14 MeSH descriptor: [Diabetes Mellitus, Type 2] explode all trees  #15 (“Diabetes Mellitus, Type 2” OR “Diabetes Mellitus, Noninsulin-Dependent” OR “Diabetes Mellitus, Non Insulin Dependent” OR “Diabetes Mellitus, Non-Insulin-Dependent” OR “Non-Insulin-Dependent Diabetes Mellitus” OR “Diabetes Mellitus, Stable” OR “Stable Diabetes Mellitus” OR “Diabetes Mellitus, Type II” OR “NIDDM” OR “Diabetes Mellitus, Noninsulin Dependent” OR “Diabetes Mellitus, Slow-Onset” OR “Diabetes Mellitus, Slow Onset” OR “Slow-Onset Diabetes Mellitus” OR “Type 2 Diabetes Mellitus” OR “Noninsulin-Dependent Diabetes Mellitus” OR “Noninsulin Dependent Diabetes Mellitus” OR “Type 2 Diabetes” OR “Diabetes, Type 2”):ti,ab,kw   #16 (#1 OR #2 OR #3 OR #4 OR #5 OR #6 OR #7 OR #8 OR #9 OR #10 OR #11 OR #12 OR #13) AND (#14 OR #15)  #17 ("Randomized Controlled Trial" OR "Clinical Trial" OR "Clinical Trial, Phase III" OR "Clinical Trial, Phase II"):pt  #18 MeSH descriptor: [Randomized Controlled Trials as Topic] explode all trees  #19 MeSH descriptor: [Clinical Trials as Topic] explode all trees  #20 MeSH descriptor: [Double-Blind Method] explode all trees  #21 MeSH descriptor: [Random Allocation] explode all trees  #22 MeSH descriptor: [Random Allocation] explode all trees  #23 MeSH descriptor: [Placebos] explode all trees  #24 MeSH descriptor: [Cross-Over Studies] explode all trees  #25 (random* OR placebo*):ti,ab,kw  #26 (((randomized OR randomised OR clinical OR control*) AND trial)):ti,ab,kw  #27 (((singl* OR doubl* OR trebl* OR tripl*) AND (blind* OR mask*))):ti,ab,kw  #28 (((crossover OR cross-over OR “cross over”) AND (design* OR study OR studies OR procedure* OR trial*))):ti,ab,kw  #29 #16 AND (#17 OR #18 OR #19 OR #20 OR #21 OR #22 OR #23 OR #24 OR #25 OR #26 OR #27 OR #28) | 4622 |
| **Google Scholar** | (“Exercise” OR “Ejercicio” OR “aerobic training” OR “ejercicio aeróbico” OR “Sport” OR “Deporte” OR “Walking” OR “caminata OR “running” OR “carrera”) AND (“Diabetes mellitus type 2” OR “Type 2 diabetes”) | 200 primeros resultados |

**Material suplementario 2**. Lista de estudios excluidos luego de revisión a texto completo y no accedidos

| **CITA** | **PRIMER AUTOR** | **TITULO** | **AÑO** | **RAZON DE EXCLUSION** |
| --- | --- | --- | --- | --- |
| 1 | Serin | The Effect of the Transtheoretical Model Based Walking Exercise Training and Follow-Up on Improving Exercise Behavior and Metabolic Control in Patients with Type 2 Diabetes | 2020 | not elderly patients |
| 2 | Adeniyi | Neuromusculoskeletal disorders in patients with type 2 diabetes mellitus: Outcome of a twelve-week therapeutic exercise programme | 2010 | not elderly patients |
| 3 | Albu | Metabolic changes following a 1-year diet and exercise intervention in patients with type 2 diabetes | 2010 | not elderly patients |
| 4 | Balducci | Anti-inflammatory effect of exercise training in subjects with type 2 diabetes and the metabolic syndrome is dependent on exercise modalities and independent of weight loss | 2010 | not elderly patients |
| 5 | Chen | Effect of T'ai Chi exercise on biochemical profiles and oxidative stress indicators in obese patients with type 2 diabetes | 2010 | not elderly patients |
| 6 | Gram | Effects of nordic walking and exercise in type 2 diabetes mellitus: A randomized controlled trial | 2010 | not elderly patients |
| 7 | Kadoglou | Exercise ameliorates serum MMP-9 and TIMP-2 levels in patients with type 2 diabetes | 2010 | not elderly patients |
| 8 | Koo | The effects of total energy expenditure from all levels of physical activity vs. physical activity energy expenditure from moderate-to-vigorous activity on visceral fat and insulin sensitivity in obese Type 2 diabetic women | 2010 | not elderly patients |
| 9 | Larose | Effect of exercise training on physical fitness in type II diabetes mellitus | 2010 | not elderly patients |
| 10 | Meex | Restoration of muscle mitochondrial function and metabolic flexibility in type 2 diabetes by exercise training is paralleled by increased myocellular fat storage and improved insulin sensitivity | 2010 | without control group |
| 11 | Negri | Supervised walking groups to increase physical activity in type 2 diabetic patients | 2010 | not elderly patients |
| 12 | Reid | Effects of aerobic exercise, resistance exercise or both, on patient-reported health status and well-being in type 2 diabetes mellitus: A randomised trial | 2010 | not elderly patients |
| 13 | Sixt | Long- but not short-term multifactorial intervention with focus on exercise training improves coronary endothelial dysfunction in diabetes mellitus type 2 and coronary artery disease | 2010 | not elderly patients |
| 14 | Solomon | Improved pancreatic β-cell function in type 2 diabetic patients after lifestyle-induced weight loss is related to glucose-dependent insulinotropic polypeptide | 2010 | without control group |
| 15 | Yavari | The effect of aerobic exercise on glycosylated hemoglobin values in type 2 diabetes patients | 2010 | not elderly patients |
| 16 | Andrews | Diet or diet plus physical activity versus usual care in patients with newly diagnosed type 2 diabetes: the Early ACTID randomised controlled trial | 2011 | not elderly patients |
| 17 | Backx | The effect of a supported exercise programme in patients with newly diagnosed Type 2 diabetes: a pilot study | 2011 | not elderly patients |
| 18 | Behboudi | Effects of aerobic exercise and whole body vibration on glycaemia control in type 2 diabetic males | 2011 | not elderly patients |
| 19 | Bello | Effects of aerobic exercise on selected physiological parameters and quality of life in patients with type 2 diabetes mellitus | 2011 | not elderly patients |
| 20 | Ferrer | [Benefits of a home-based physical exercise program in elderly subjects with type 2 diabetes mellitus] | 2011 | not intervention |
| 21 | Hare | Application of an Exercise Intervention on the Evolution of Diastolic Dysfunction in Patients With Diabetes Mellitus Efficacy and Effectiveness | 2011 | not elderly patients |
| 22 | Huebschmann | Exercise attenuates the premature cardiovascular aging effects of type 2 diabetes mellitus | 2011 | not clinical trial |
| 23 | Jorge | The effects of aerobic, resistance, and combined exercise on metabolic control, inflammatory markers, adipocytokines, and muscle insulin signaling in patients with type 2 diabetes mellitus | 2011 | not elderly patients |
| 24 | Kurban | Effect of Chronic Regular Exercise on Serum Ischemia-Modified Albumin Levels and Oxidative Stress in Type 2 Diabetes Mellitus | 2011 | not elderly patients |
| 25 | Larose | Associations between physical fitness and HbA(1c) in type 2 diabetes mellitus | 2011 | not elderly patients |
| 26 | Lucotti | Aerobic and resistance training effects compared to aerobic training alone in obese type 2 diabetic patients on diet treatment | 2011 | not elderly patients |
| 27 | Moghaddam | Training increases peroxiredoxin 2 contents in the erythrocytes of overweight/obese men suffering from type 2 diabetes | 2011 | not elderly patients |
| 28 | Nicolucci | Improvement of quality of life with supervised exercise training in subjects with type 2 diabetes mellitus | 2011 | not clinical trial |
| 29 | Schneider | Design and methods for a pilot randomized clinical trial involving exercise and behavioral activation to treat comorbid type 2 diabetes and major depressive disorder | 2011 | not elderly patients |
| 30 | Schultz | Lifestyle change diminishes a hypertensive response to exercise in type 2 diabetes | 2011 | not elderly patients |
| 31 | Snel | Immediate and long-term effects of addition of exercise to a 16-week very low calorie diet on low-grade inflammation in obese, insulin-dependent type 2 diabetic patients | 2011 | not elderly patients |
| 32 | Stewart | Exercise Training Fails to Reduce Blood Pressure in Type 2 Diabetes: A Randomized Controlled Trial Stewart | 2011 | not clinical trial |
| 33 | Teixeira | Regular physical exercise training assists in preventing type 2 diabetes development: focus on its antioxidant and anti-inflammatory properties | 2011 | not clinical trial |
| 34 | Aguila | Efecto del ejercicio físico en el control metabólico y en factores de riesgo de pacientes con diabetes mellitus tipo 2: estudio cuasi experimental | 2012 | not elderly patients |
| 35 | Ahn | Effects of Tai Chi Exercise on glucose control, neuropathy scores, balance, and quality of life in patients with type 2 diabetes and neuropathy | 2012 | not elderly patients |
| 36 | Balducci | Changes in physical fitness predict improvements in modifiable cardiovascular risk factors independently of body weight loss in subjects with type 2 diabetes participating in the Italian Diabetes and Exercise Study (IDES) | 2012 | not elderly patients |
| 37 | Barone | A randomized trial of exercise for blood pressure reduction in type 2 diabetes: effect on flow-mediated dilation and circulating biomarkers of endothelial function | 2012 | not elderly patients |
| 38 | Choi | Effects of exercise on sRAGE levels and cardiometabolic risk factors in patients with type 2 diabetes: a randomized controlled trial | 2012 | not elderly patients |
| 39 | Cooper | Sedentary time, breaks in sedentary time and metabolic variables in people with newly diagnosed type 2 diabetes | 2012 | not elderly patients |
| 40 | de Oliveira | The effect of different training programs on antioxidant status, oxidative stress, and metabolic control in type 2 diabetes | 2012 | not elderly patients |
| 41 | Dobrosielski | Effect of exercise on blood pressure in type 2 diabetes: a randomized controlled trial | 2012 | not elderly patients |
| 42 | Florez | Impact of lifestyle intervention and metformin on health-related quality of life: the diabetes prevention program randomized trial | 2012 | not elderly patients |
| 43 | Gallagher | A randomised trial of a weight loss intervention for overweight and obese people diagnosed with coronary heart disease and/or type 2 diabetes | 2012 | not elderly patients |
| 44 | Gillen | Acute high‐intensity interval exercise reduces the postprandial glucose response and prevalence of hyperglycaemia in patients with type 2 diabetes | 2012 | no outcome |
| 45 | Hajihasani, | Effects of eccentric and concentric exercises on some blood biochemical parameters in patients with type 2 diabetes | 2012 | not elderly patients |
| 46 | Lijima | Lower physical activity is a strong predictor of cardiovascular events in elderly patients with type 2 diabetes mellitus beyond traditional risk factors: the Japanese Elderly Diabetes Intervention Trial | 2012 | not clinical trial |
| 47 | Jung | Effects of aerobic exercise intensity on abdominal and thigh adipose tissue and skeletal muscle attenuation in overweight women with type 2 diabetes mellitus | 2012 | not elderly patients |
| 48 | Kadoglou | The impact of aerobic exercise training on novel adipokines, apelin and ghrelin, in patients with type 2 diabetes | 2012 | not elderly patients |
| 49 | Karstoft | The effects and physiological mechanisms of free-living interval-walking training on glycaemic control in type 2 diabetes patients: a randomised, controlled trial | 2012 | not elderly patients |
| 50 | Mackenzie | Intermittent exercise with and without hypoxia improves insulin sensitivity in individuals with type 2 diabetes | 2012 | not elderly patients |
| 51 | Marquez | El ejercicio en el tratamiento de la diabetes mellitus tipo 2 | 2012 | not clinical trial |
| 52 | Nitert | Impact of an exercise intervention on DNA methylation in skeletal muscle from first-degree relatives of patients with type 2 diabetes | 2012 | not elderly patients |
| 53 | Sazlina | Promoting physical activity in sedentary elderly Malays with type 2 diabetes: a protocol for randomised controlled trial | 2012 | not clinical trial |
| 54 | Shaw | Prolonged exercise training increases intramuscular lipid content and perilipin 2 expression in type I muscle fibers of patients with type 2 diabetes | 2012 | without control group |
| 55 | Simmonds | Preliminary findings in the heart rate variability and haemorheology response to varied frequency and duration of walking in women 65-74 yr with type 2 diabetes | 2012 | without control group |
| 56 | Snel | Effects of adding exercise to a 16-week very low-calorie diet in obese, insulin-dependent type 2 diabetes mellitus patients | 2012 | not elderly patients |
| 57 | Snel | Quality of life in type 2 diabetes mellitus after a very low calorie diet and exercise | 2012 | no outcome |
| 58 | Swift | Effect of exercise training modality on C-reactive protein in type 2 diabetes | 2012 | not elderly patients |
| 59 | Swift | The effect of exercise training modality on serum brain derived neurotrophic factor levels in individuals with type 2 diabetes | 2012 | not elderly patients |
| 60 | Teixeira | Regular physical exercise as a strategy to improve antioxidant and anti-inflammatory status: benefits in type 2 diabetes mellitus | 2012 | not clinical trial |
| 61 | Van der Heijden | Testing the effectiveness of a self-efficacy based exercise intervention for inactive people with type 2 diabetes mellitus: design of a controlled clinical trial | 2012 | not elderly patients |
| 62 | Van dijk | Both resistance- and endurance-type exercise reduce the prevalence of hyperglycaemia in individuals with impaired glucose tolerance and in insulin-treated and non-insulin-treated type 2 diabetic patients | 2012 | not intervention |
| 63 | Yavari | EFFECT OF AEROBIC EXERCISE, RESISTANCE TRAINING OR COMBINED TRAINING ON GLYCAEMIC CONTROL AND CARDIOVASCULAR RISK FACTORS IN PATIENTS WITH TYPE 2 DIABETES | 2012 | not elderly patients |
| 64 | Abd | Impact of moderate versus mild aerobic exercise training on inflammatory cytokines in obese type 2 diabetic patients: a randomized clinical trial | 2013 | not elderly patients |
| 65 | Adeniyi | Time course of improvement of metabolic parameters after a 12 week physical exercise programme in patients with type 2 diabetes: the influence of gender in a Nigerian population | 2013 | not elderly patients |
| 66 | Ahmad | Influence of 3 months endurance training on red cell deformability in non insulin dependent type 2 diabetes mellitus patients | 2013 | not elderly patients |
| 67 | Barakat | Changes in physical activity and modelled cardiovascular risk following diagnosis of diabetes: 1-year results from the ADDITION-Cambridge trial cohort | 2013 | not elderly patients |
| 68 | Beverly | Impact of Reinforcement of Diabetes Self-Care on Poorly Controlled Diabetes A Randomized Controlled Trial | 2013 | not elderly patients |
| 69 | Blomster | The influence of physical activity on vascular complications and mortality in patients with type 2 diabetes mellitus | 2013 | no outcome |
| 70 | de la Torre | Early management of type 2 diabetes based on a SMBG strategy: the way to diabetes regression-the St Carlos study | 2013 | not elderly patients |
| 71 | Dobrosielski | Effect of exercise on abdominal fat loss in men and women with and without type 2 diabetes | 2013 | not elderly patients |
| 72 | Espeland | Intensive weight loss intervention in older individuals: results from the Action for Health in Diabetes Type 2 diabetes mellitus trial | 2013 | not intervention |
| 73 | Fiocco | The effects of an exercise and lifestyle intervention program on cardiovascular, metabolic factors and cognitive performance in middle-aged adults with type ii diabetes: A pilot study | 2013 | not elderly patients |
| 74 | Foster | A randomized comparison of a commercially available portion-controlled weight-loss intervention with a diabetes self-management education program | 2013 | not elderly patients |
| 75 | Fritz | Effects of Nordic walking on cardiovascular risk factors in overweight individuals with type 2 diabetes, impaired or normal glucose tolerance | 2013 | not elderly patients |
| 76 | de la Torre | Early management of type 2 diabetes based on a SMBG strategy: The way to diabetes regression - The St Carlos study: A 3-year, prospective, randomized, clinic-based, interventional study with parallel groups | 2013 | not elderly patients |
| 77 | Jakicic | Four-year change in cardiorespiratory fitness and influence on glycemic control in adults with type 2 diabetes in a randomized trial: the Look AHEAD Trial | 2013 | not elderly patients |
| 78 | Johannsen | Determinants of the Changes in Glycemic Control with Exercise Training in Type 2 Diabetes: A Randomized Trial | 2013 | not elderly patients |
| 79 | Karstoft | The Effects of Free-Living Interval Walking Training on Glycemic Control, Body Composition, and Physical Fitness in Type 2 Diabetic Patients A randomized, controlled trial | 2013 | not elderly patients |
| 80 | Liu | The effect of Tai Chi on health-related quality of life in people with elevated blood glucose or diabetes: A randomized controlled trial | 2013 | not elderly patients |
| 81 | Mavros | Changes in insulin resistance and HbA1c are related to exercise-mediated changes in body composition in older adults with type 2 diabetes: interim outcomes from the GREAT2DO trial | 2013 | not intervention |
| 82 | Montero | Effects of exercise training on arterial function in type 2 diabetes mellitus | 2013 | not clinical trial |
| 83 | Mueller | Weight-bearing versus nonweight-bearing exercise for persons with diabetes and peripheral neuropathy: A randomized controlled trial | 2013 | not elderly patients |
| 84 | Saha | A culturally adapted lifestyle intervention addressing a Middle Eastern immigrant population at risk of diabetes, the MEDIM (impact of Migration and Ethnicity on Diabetes In Malmö): study protocol for a randomized controlled trial | 2013 | not clinical trial |
| 85 | Senechal | Changes in body fat distribution and fitness are associated with changes in hemoglobin A1c after 9 months of exercise training: results from the HART-D study | 2013 | not elderly patients |
| 86 | Solomon | The influence of hyperglycemia on the therapeutic effect of exercise on glycemic control in patients with type 2 diabetes mellitus | 2013 | not elderly patients |
| 87 | Sukala | Exercise improves quality of life in indigenous Polynesian peoples with type 2 diabetes and visceral obesity | 2013 | not elderly patients |
| 88 | Terada | Feasibility and preliminary efficacy of high intensity interval training in type 2 diabetes | 2013 | without control group |
| 89 | Terada | Exploring the variability in acute glycemic responses to exercise in type 2 diabetes | 2013 | without control group |
| 90 | Tomar | Effects of a 12-week aerobic training on glycemic control in type 2 diabetes mellitus male patients | 2013 | not elderly patients |
| 91 | Wenning | Endurance exercise alters cellular immune status and resistin concentrations in men suffering from non-insulin-dependent type 2 diabetes | 2013 | not elderly patients |
| 92 | Arslan | Effect of Aerobic Exercise Training on MDA and TNF- α Levels in Patients with Type 2 Diabetes Mellitus | 2014 | not elderly patients |
| 93 | Avery | Movement as Medicine for Type 2 Diabetes: protocol for an open pilot study and external pilot clustered randomised controlled trial to assess acceptability, feasibility and fidelity of a multifaceted behavioural intervention targeting physical activity in primary care | 2014 | not clinical trial |
| 94 | Balducci | Physical exercise as therapy for type 2 diabetes mellitus | 2014 | not clinical trial |
| 95 | Bello | The Effect of a Long-Term, Community-Based Exercise Program on Bone Mineral Density in Postmenopausal Women with Pre-Diabetes and Type 2 Diabetes | 2014 | not elderly patients |
| 96 | Clarke | The INSPIRED study: A randomised controlled trial of the Whole Person Model of disease self-management for people with type 2 diabetes | 2014 | not elderly patients |
| 97 | Cooper | Association between objectively assessed sedentary time and physical activity with metabolic risk factors among people with recently diagnosed type 2 diabetes | 2014 | not elderly patients |
| 98 | Dasgupta | Step Monitoring to improve ARTERial health (SMARTER) through step count prescription in type 2 diabetes and hypertension: Trial design and methods | 2014 | not clinical trial |
| 99 | de sousa | Positive effects of football on fitness, lipid profile, and insulin resistance in Brazilian patients with type 2 diabetes | 2014 | not elderly patients |
| 100 | Earnest | Aerobic and strength training in concomitant metabolic syndrome and type 2 diabetes | 2014 | not elderly patients |
| 101 | Espeland | Long-term impact of behavioral weight loss intervention on cognitive function | 2014 | not elderly patients |
| 102 | Falconer | Sedentary time and markers of inflammation in people with newly diagnosed type 2 diabetes | 2014 | not elderly patients |
| 103 | Gallagher | Changes in adipose tissue depots and metabolic markers following a 1-year diet and exercise intervention in overweight and obese patients with type 2 diabetes | 2014 | not elderly patients |
| 104 | Gibbs | Effect of improved fitness beyond weight loss on cardiovascular risk factors in individuals with type 2 diabetes in the Look AHEAD study | 2014 | not elderly patients |
| 105 | Griffin | Multiple behaviour change intervention and outcomes in recently diagnosed type 2 diabetes: the ADDITION-Plus randomised controlled trial | 2014 | not elderly patients |
| 106 | Guglani | Effect of progressive pedometer based walking intervention on quality of life and general well being among patients with type 2 diabetes | 2014 | not elderly patients |
| 107 | Hollekim | High-intensity interval exercise effectively improves cardiac function in patients with type 2 diabetes mellitus and diastolic dysfunction: a randomized controlled trial | 2014 | not elderly patients |
| 108 | Kaizu | Impact of leisure-time physical activity on glycemic control and cardiovascular risk factors in Japanese patients with type 2 diabetes mellitus: The Fukuoka Diabetes Registry | 2014 | not elderly patients |
| 109 | Kim | Effect of different training mode on Interleukin-6 (IL-6) and C-reactive protein (CRP) in type 2 diabetes mellitus (T2DM) patients | 2014 | not elderly patients |
| 110 | Krause | The effects of aerobic exercise training at two different intensities in obesity and type 2 diabetes: implications for oxidative stress, low-grade inflammation and nitric oxide production | 2014 | not elderly patients |
| 111 | Lee | The Effects of 12 Weeks Regular Aerobic Exercise on Brain-derived Neurotrophic Factor and Inflammatory Factors in Juvenile Obesity and Type 2 Diabetes Mellitus | 2014 | not elderly patients |
| 112 | Mangeri | A standard ballroom and Latin dance program to improve fitness and adherence to physical activity in individuals with type 2 diabetes and in obesity | 2014 | not elderly patients |
| 113 | Mensberg | Near-Normalization of Glycemic Control in Patients with Type 2 Diabetes with a Glucagon-like Peptide-1 Receptor Agonist in Combination with Exercise Training: A Randomized, Double-Blinded, Placebo-Controlled Clinical Trial | 2014 | without control group |
| 114 | Mitranum | Continuous vs interval training on glycemic control and macro- and microvascular reactivity in type 2 diabetic patients | 2014 | not elderly patients |
| 115 | Morrison | Exercise improves gait, reaction time and postural stability in older adults with type 2 diabetes and neuropathy | 2014 | not elderly patients |
| 116 | Phillips | A comparison of the effects of aerobic and intense exercise on the type 2 diabetes mellitus risk marker adipokines, adiponectin and retinol binding protein-4 | 2014 | not clinical trial |
| 117 | Sacre | A six-month exercise intervention in subclinical diabetic heart disease: effects on exercise capacity, autonomic and myocardial function | 2014 | not elderly patients |
| 118 | Singleton | Exercise increases cutaneous nerve density in diabetic patients without neuropathy | 2014 | not elderly patients |
| 119 | Subramanian | Effects of Physioball exercises on glycemic control and quality life of Type II diabetic patients | 2014 | not elderly patients |
| 120 | Thompson | Effect of diet or diet plus physical activity versus usual care on inflammatory markers in patients with newly diagnosed type 2 diabetes: the Early ACTivity in Diabetes (ACTID) randomized, controlled trial | 2014 | not elderly patients |
| 121 | Yan | Effect of Aerobic Training on Glucose Control and Blood Pressure in T2DDM East African Males | 2014 | not elderly patients |
| 122 | Ades | Remission of Recently Diagnosed Type 2 Diabetes Mellitus With Weight Loss and Exercise | 2015 | not elderly patients |
| 123 | Ades | A lifestyle program of exercise and weight loss is effective in preventing and treating type 2 diabetes mellitus: why are programs not more available? | 2015 | not clinical trial |
| 124 | Al Shreef | Bone metabolism and hand grip strength response to aerobic versus resistance exercise training in non-insulin dependent diabetic patients | 2015 | not elderly patients |
| 125 | Balducci | Volume-dependent effect of supervised exercise training on fatty liver and visceral adiposity index in subjects with type 2 diabetes The Italian Diabetes Exercise Study (IDES) | 2015 | not elderly patients |
| 126 | Beavers | Body weight dynamics following intentional weight loss and physical performance: the Look AHEAD Movement and Memory Study | 2015 | not elderly patients |
| 127 | Byrkjeland | Effects of exercise training on HbA1c and VO2peak in patients with type 2 diabetes and coronary artery disease: A randomised clinical trial | 2015 | not elderly patients |
| 128 | Climie | Exercise excess pressure and exercise-induced albuminuria in patients with type 2 diabetes mellitus | 2015 | not elderly patients |
| 129 | da Silva | Impact of the activation of intention to perform physical activity in type II diabetics: a randomized clinical trial | 2015 | not elderly patients |
| 130 | Dede | Influence of Exercise on Leptin, Adiponectin and Quality of Life in Type 2 Diabetics | 2015 | not elderly patients |
| 131 | Emerenziani | Effects of Aerobic Exercise Based upon Heart Rate at Aerobic Threshold in Obese Elderly Subjects with Type 2 Diabetes | 2015 | not elderly patients |
| 132 | Fex | Effect of Elliptical High Intensity Interval Training on Metabolic Risk Factor in Pre- and Type 2 Diabetes Patients: A Pilot Study | 2015 | not elderly patients |
| 133 | Green | Cardiovascular control during exercise in type 2 diabetes mellitus | 2015 | not clinical trial |
| 134 | Hamasaki | Daily physical activity assessed by a triaxial accelerometer is beneficially associated with waist circumference, serum triglycerides, and insulin resistance in Japanese patients with prediabetes or untreated early type 2 diabetes | 2015 | not elderly patients |
| 135 | Higgins | Heterogeneous responses of personalised high intensity interval training on type 2 diabetes mellitus and cardiovascular disease risk in young healthy adults | 2015 | not elderly patients |
| 136 | Johnson | Increase in Daily Steps After an Exercise Specialist Led Lifestyle Intervention for Adults With Type 2 Diabetes In Primary Care: A Controlled Implementation Trial | 2015 | not elderly patients |
| 137 | Jung | Comparison of the effects of Korean mindfulness-based stress reduction, walking, and patient education in diabetes mellitus | 2015 | not elderly patients |
| 138 | Kasumov | Improved insulin sensitivity after exercise training is linked to reduced plasma C14:0 ceramide in obesity and type 2 diabetes | 2015 | not elderly patients |
| 139 | Kluding | Safety of aerobic exercise in people with diabetic peripheral neuropathy: single-group clinical trial | 2015 | not elderly patients |
| 140 | Lee | An investigation and comparison of the effectiveness of different exercise programmes in improving glucose metabolism and pancreatic β cell function of type 2 diabetes patients | 2015 | not elderly patients |
| 141 | Maharaj | The effect of rebound exercise and treadmill walking on the quality of life for patients with non-insulin-dependent type 2 diabetes | 2015 | not elderly patients |
| 142 | Mobasseri | Effect of a long-term regular physical activity on hypertension and body mass index in type 2 diabetes patients | 2015 | not elderly patients |
| 143 | Njerve | Effects of 12 months exercise intervention on adipose tissue expression of chemokines in patients with type 2 diabetes and stable coronary artery disease: a substudy of a randomized controlled trial (RCT) | 2015 | not elderly patients |
| 144 | Nylen | Enhanced fitness and renal function in Type 2 diabetes | 2015 | not elderly patients |
| 145 | Olson | Impact of a brief intervention on self-regulation, self-efficacy and physical activity in older adults with type 2 diabetes | 2015 | not elderly patients |
| 146 | Poblete | Effects of high intensity interval training versus moderate intensity continuous training on the reduction of oxidative stress in type 2 diabetic adult patients: CAT | 2015 | not elderly patients |
| 147 | Ring | Influence of physical activity and gender on arterial function in type 2 diabetes, normal and impaired glucose tolerance | 2015 | not elderly patients |
| 148 | Senechal | Association between Changes in Muscle Quality with Exercise Training and Changes in Cardiorespiratory Fitness Measures in Individuals with Type 2 Diabetes Mellitus: Results from the HART-D Study | 2015 | not elderly patients |
| 149 | Sentinelli | Positive effects of Nordic Walking on anthropometric and metabolic variables in women with type 2 diabetes mellitus | 2015 | not elderly patients |
| 150 | Stegen | Aerobic and resistance training do not influence plasma carnosinase content or activity in type 2 diabetes | 2015 | not elderly patients |
| 151 | Stephens | Resistance to the beneficial effects of exercise in type 2 diabetes: are some individuals programmed to fail? | 2015 | not elderly patients |
| 152 | Subramanian | Aerobic exercises on type II diabetic with hypertension | 2015 | not elderly patients |
| 153 | Teychenne | Adoption and maintenance of gym-based strength training in the community setting in adults with excess weight or type 2 diabetes: a randomized controlled trial | 2015 | not elderly patients |
| 154 | Tsai | Brain-derived neurotrophic factor correlated with muscle strength in subjects undergoing stationary bicycle exercise training | 2015 | not elderly patients |
| 155 | Alghadir | Cellular fibronectin response to supervised moderate aerobic training in patients with type 2 diabetes | 2016 | not elderly patients |
| 156 | Assad | Effectiveness of a Lifestyle Intervention in Patients with Type 2 Diabetes: The Physical Activity and Nutrition for Diabetes in Alberta (PANDA) Trial | 2016 | not elderly patients |
| 157 | Bener | The role of vitamin D, obesity and physical exercise in regulation of glycemia in Type 2 Diabetes Mellitus patients | 2016 | not elderly patients |
| 158 | Bentley | Feasibility study of portable technology for weight loss and HbA1c control in type 2 diabetes | 2016 | not elderly patients |
| 159 | Cassidy | High intensity intermittent exercise improves cardiac structure and function and reduces liver fat in patients with type 2 diabetes: a randomised controlled trial | 2016 | not elderly patients |
| 160 | Cox | Glycemic load, exercise, and monitoring blood glucose (GEM): A paradigm shift in the treatment of type 2 diabetes mellitus | 2016 | not elderly patients |
| 161 | Dadgostar | Supervised group-exercise therapy versus home-based exercise therapy: Their effects on Quality of Life and cardiovascular risk factors in women with type 2 diabetes | 2016 | not elderly patients |
| 162 | Deguchi | Acute effect of fast walking on postprandial blood glucose control in type 2 diabetes | 2016 | not elderly patients |
| 163 | Delevatti | Glucose control can be similarly improved after aquatic or dry-land aerobic training in patients with type 2 diabetes: A randomized clinical trial | 2016 | not elderly patients |
| 164 | Delevatti | Glycemic reductions following water- and land-based exercise in patients with type 2 diabetes mellitus | 2016 | not elderly patients |
| 165 | Gamiochipi | Effect of an intensive metabolic control lifestyle intervention in type-2 diabetes patients | 2016 | not elderly patients |
| 166 | Hollekim | Exercise Training Normalizes Timing of Left Ventricular Untwist Rate, but Not Peak Untwist Rate, in Individuals with Type 2 Diabetes and Diastolic Dysfunction: A Pilot Study | 2016 | not elderly patients |
| 167 | Maillard | High-intensity interval training reduces abdominal fat mass in postmenopausal women with type 2 diabetes | 2016 | without control group |
| 168 | Mehdizadeh | Investigation of Plasma Visfatin Changes in Women with Type 2 Diabetes followed by Endurance, Resistance and Combined Exercise: The Role of Lipid Profile, Glycemic Indices and Insulin Resistance | 2016 | not elderly patients |
| 169 | Miyauchi | Exercise Therapy for Management of Type 2 Diabetes Mellitus: Superior Efficacy of Activity Monitors over Pedometers | 2016 | not elderly patients |
| 170 | Mousavian | Changes in some of the indicators of liver inflammation and cardiovascular risk factors during a term of synthetic aerobic exercise of diabetic women | 2016 | not elderly patients |
| 171 | Myette | The Effect of Exercise with or Without Metformin on Glucose Profiles in Type 2 Diabetes: A Pilot Study | 2016 | not elderly patients |
| 172 | Picone | SIX MONTHS EXERCISE INTERVENTION SIGNIFICANTLY IMPROVES ALBUMIN-CREATININE RATIO IN PATIENTS WITH TYPE 2 DIABETES: A RANDOMISED CONTROLLED TRIAL | 2016 | not intervention |
| 173 | Revdal | Can Time Efficient Exercise Improve Cardiometabolic Risk Factors in Type 2 Diabetes? A Pilot Study | 2016 | not elderly patients |
| 174 | Sobhanipour | A comparative study on the effect of endurance and resistance training on angiogenesis factors in elderly diabetic patients | 2016 | not elderly patients |
| 175 | Steidle | Does exercise training impact clock genes in patients with coronary artery disease and type 2 diabetes mellitus? | 2016 | not elderly patients |
| 176 | Terada | Targeting specific interstitial glycemic parameters with high-intensity interval exercise and fasted-state exercise in type 2 diabetes | 2016 | not elderly patients |
| 177 | Wagner | Improvement of insulin sensitivity in response to exercise training in type 2 diabetes mellitus is associated with vascular endothelial growth factor A expression | 2016 | not elderly patients |
| 178 | AminiLari | The Effect of 12 Weeks Aerobic, Resistance, and Combined Exercises on Omentin-1 Levels and Insulin Resistance among Type 2 Diabetic Middle-Aged Women | 2017 | not elderly patients |
| 179 | Bellia | Exercise individualized by TRIMPi method reduces arterial stiffness in early onset type 2 diabetic patients: A randomized controlled trial with aerobic interval training | 2017 | not elderly patients |
| 180 | Bratseth | Procoagulant activity in patients with combined type 2 diabetes and coronary artery disease: No effects of long-term exercise training | 2017 | not elderly patients |
| 181 | Byrkjeland | Reduced endothelial activation after exercise is associated with improved HbA(1c) in patients with type 2 diabetes and coronary artery disease | 2017 | not elderly patients |
| 182 | Carrasquillo | Effect of a Community Health Worker Intervention Among Latinos With Poorly Controlled Type 2 Diabetes: The Miami Healthy Heart Initiative Randomized Clinical Trial | 2017 | not elderly patients |
| 183 | Dasgupta | Physician step prescription and monitoring to improve ARTERial health (SMARTER): A randomized controlled trial in patients with type 2 diabetes and hypertension | 2017 | not elderly patients |
| 184 | Do valle Nascimento | A pilot study of a Community Health Agent-led type 2 diabetes self-management program using Motivational Interviewing-based approaches in a public primary care center in São Paulo, Brazil | 2017 | not elderly patients |
| 185 | dos anjos | Effects of aerobic exercise on functional capacity, anthropometric measurements and inflammatory markers in diabetic elderly women | 2017 | without control group |
| 186 | Espeland | Effects of Physical Activity Intervention on Physical and Cognitive Function in Sedentary Adults With and Without Diabetes | 2017 | not elderly patients |
| 187 | Francois | Combined Interval Training and Post-exercise Nutrition in Type 2 Diabetes: A Randomized Control Trial | 2017 | not elderly patients |
| 188 | Francois | The impact of acute high-intensity interval exercise on biomarkers of cardiovascular health in type 2 diabetes | 2017 | not elderly patients |
| 189 | Gallagher | Changes in skeletal muscle and organ size after a weight-loss intervention in overweight and obese type 2 diabetic patients | 2017 | not elderly patients |
| 190 | Galle | Improving Self-Management of Type 2 Diabetes in Overweight and Inactive Patients Through an Educational and Motivational Intervention Addressing Diet and Physical Activity: A Prospective Study in Naples, South Italy | 2017 | not elderly patients |
| 191 | Jiang | The effect of care intervention for obese patients with type II diabetes | 2017 | not elderly patients |
| 192 | Karimi | Effects of Supervised Structured Aerobic Exercise Training Program on Interleukin-6, Nitric Oxide Synthase-1, and Cyclooxygenase-2 in Type 2 Diabetes Mellitus | 2017 | not elderly patients |
| 193 | Kaur | Optimization of aerobic exercise protocols in diabetes mellitus: A randomized trial | 2017 | not elderly patients |
| 194 | Liu | Effect of square aerobic exercise on cardiovascular risk factors and health-related quality of life in Chinese women with type 2 diabetes | 2017 | not elderly patients |
| 195 | Mallard | Exercise intensity, redox homeostasis and inflammation in type 2 diabetes mellitus | 2017 | not elderly patients |
| 196 | Mazzuca | Supervised vs. self-selected physical activity for individuals with diabetes and obesity: the Lifestyle Gym program | 2017 | not elderly patients |
| 197 | Miele | The effects of chronic aerobic exercise on cardiovascular risk factors in persons with diabetes mellitus | 2017 | not clinical trial |
| 198 | Miyamoto | Non-locomotive physical activity intervention using a tri-axial accelerometer reduces sedentary time in type 2 diabetes | 2017 | not elderly patients |
| 199 | Najafipour | Effect of regular exercise training on changes in HbA1c, BMI and VO(2)max among patients with type 2 diabetes mellitus: an 8-year trial | 2017 | not elderly patients |
| 200 | Nygaard | Long-term effects of daily postprandial physical activity on blood glucose: a randomized controlled trial | 2017 | not elderly patients |
| 201 | Pahra | Impact of post-meal and one-time daily exercise in patient with type 2 diabetes mellitus: a randomized crossover study | 2017 | not elderly patients |
| 202 | Pandey | The Impact of Burst Exercise on Cardiometabolic Status of Patients Newly Diagnosed With Type 2 Diabetes | 2017 | not elderly patients |
| 203 | Shakil | Effects of supervised structured aerobic exercise training program on high and low density lipoprotein in patients with type II diabetes mellitus | 2017 | not elderly patients |
| 204 | Shakil | Effects of supervised structured aerobic exercise training program on fasting blood glucose level, plasma insulin level, glycemic control, and insulin resistance in type 2 diabetes mellitus | 2017 | not elderly patients |
| 205 | Stoa | High-intensity aerobic interval training improves aerobic fitness and HbA1c among persons diagnosed with type 2 diabetes | 2017 | not elderly patients |
| 206 | Suntraluck | The Relative Efficacy of Land-Based and Water-Based Exercise Training on Macro- and Microvascular Functions in Older Patients With Type 2 Diabetes | 2017 | without control group |
| 207 | Terada | Cardiometabolic risk factors in type 2 diabetes with high fat and low muscle mass: At baseline and in response to exercise | 2017 | not elderly patients |
| 208 | Rehman | Effects of supervised structured aerobic exercise training programme on level of Exertion, dyspnoea, VO2 max and Body Mass Index in patients with type 2 diabetes mellitus | 2017 | not elderly patients |
| 209 | Vanroy | Short- and long-term effects of a need-supportive physical activity intervention among patients with type 2 diabetes mellitus: A randomized controlled pilot trial | 2017 | not elderly patients |
| 210 | Arovah | Walking with Diabetes (WW-DIAB) programme a walking programme for Indonesian type 2 diabetes mellitus patients: A pilot randomised controlled trial | 2018 | not elderly patients |
| 211 | Zadeh | Diets along with interval training regimes improves inflammatory & anti-inflammatory condition in obesity with type 2 diabetes subjects | 2018 | not elderly patients |
| 212 | Bellavere | Effects of aerobic or resistance exercise training on cardiovascular autonomic function of subjects with type 2 diabetes: A pilot study | 2018 | not elderly patients |
| 213 | Junior | Multicomponent Exercise Improves Hemodynamic Parameters and Mobility, but Not Maximal Walking Speed, Transfer Capacity, and Executive Function of Older Type II Diabetic Patients | 2018 | not elderly patients |
| 214 | Duvivier | Reducing sitting time versus adding exercise: differential effects on biomarkers of endothelial dysfunction and metabolic risk | 2018 | not elderly patients |
| 215 | Faroqi | Evaluating the clinical implementation of structured exercise: A randomized controlled trial among non-insulin dependent type II diabetics | 2018 | not elderly patients |
| 216 | Francois | Cardiovascular benefits of combined interval training and post-exercise nutrition in type 2 diabetes | 2018 | not elderly patients |
| 217 | Gholami | Effect of aerobic training on nerve conduction in men with type 2 diabetes and peripheral neuropathy: A randomized controlled trial | 2018 | not elderly patients |
| 218 | Maillard | High-intensity interval training is more effective than moderate-intensity continuous training in reducing abdominal fat mass in postmenopausal women with type 2 diabetes: A randomized crossover study | 2018 | not intervention |
| 219 | Nomura | Assessment of lower extremity muscle mass, muscle strength, and exercise therapy in elderly patients with diabetes mellitus | 2018 | not clinical trial |
| 220 | Nuhu | Influence of a mini-trampoline rebound exercise program on insulin resistance, lipid profile and central obesity in individuals with type 2 diabetes | 2018 | not elderly patients |
| 221 | Pisabarro | A 20-week exercise program improves markers of cognitive, inflammatory and metabolic status in type 2 diabetic patients | 2018 | not elderly patients |
| 222 | Delevatti | Quality of life and sleep quality are similarly improved after aquatic or dry-land aerobic training in patients with type 2 diabetes: A randomized clinical trial | 2018 | not elderly patients |
| 223 | Ranasinghe | Study protocol: a randomised controlled trial of supervised resistance training versus aerobic training in Sri Lankan adults with type 2 diabetes mellitus: SL-DART study | 2018 | not clinical trial |
| 224 | Saghebjoo | The effect of 12 weeks of aerobic training on serum levels high sensitivity C-reactive protein, tumor necrosis factor-alpha, lipid profile and anthropometric characteristics in middle-age women patients with type 2 diabetes | 2018 | not elderly patients |
| 225 | Ur | Response to a Supervised Structured Aerobic Exercise Training Program in Patients with Type 2 Diabetes Mellitus - Does Gender Make a Difference? A Randomized Controlled Clinical Trial | 2018 | not elderly patients |
| 226 | Shellington | Results From a Feasibility Study of Square-Stepping Exercise in Older AdultsWith Type 2 Diabetes and Self-Reported Cognitive Complaints to Improve Global Cognitive Functioning | 2018 | no outcome |
| 227 | Faria | COST-EFFECTIVE EXERCISE PROGRAMS ON HEALTH-STATUS OF MALAYSIAN DIABETIC INDIVIDUALS - A SOCIO-PSYCHOLOGICAL ANALYSIS | 2018 | not elderly patients |
| 228 | Verma | A dose-response study of aerobic training for oxygen uptake, oxidative stress and cardiac autonomic function in type 2 diabetes mellitus: study protocol for a randomized controlled trial | 2018 | not elderly patients |
| 229 | Winding | The effect on glycaemic control of low-volume high-intensity interval training versus endurance training in individuals with type 2 diabetes | 2018 | not elderly patients |
| 230 | Wormgoor | Acute blood glucose, cardiovascular and exaggerated responses to HIIT and moderate-intensity continuous training in men with type 2 diabetes mellitus | 2018 | not elderly patients |
| 231 | Ahmed | Effect of high intensity interval training on heart rate variability and aerobic capacity in obese adults with type 2 Diabetes Mellitus | 2019 | not elderly patients |
| 232 | Dominguez | Effect of a multifactorial intervention on the increase in physical activity in subjects with type 2 diabetes mellitus: a randomized clinical trial (EMID Study) | 2019 | not elderly patients |
| 233 | Dominguez | Acute effect of healthy walking on arterial stiffness in patients with type 2 diabetes and differences by age and sex: a pre-post intervention study | 2019 | not elderly patients |
| 234 | Bastien | Effect of PPARγ agonist on aerobic exercise capacity in relation to body fat distribution in men with type 2 diabetes mellitus and coronary artery disease: a 1-yr randomized study | 2019 | not elderly patients |
| 235 | Berger | Change in Cardiometabolic Risk Factors Associated With Magnitude of Weight Regain 3 Years After a 1-Year Intensive Lifestyle Intervention in Type 2 Diabetes Mellitus: The Look AHEAD Trial | 2019 | not elderly patients |
| 236 | Blankenship | Managing free-living hyperglycemia with exercise or interrupted sitting in type 2 diabetes | 2019 | not elderly patients |
| 237 | Bock | Reduction in HbA1c with Exercise videogames among participants with elevated HbA1c: Secondary analysis of the Wii Heart Fitness trial | 2019 | not elderly patients |
| 238 | Bourne | Electrically assisted cycling for individuals with type 2 diabetes mellitus: protocol for a pilot randomized controlled trial | 2019 | not clinical trial |
| 239 | Das | Low-energy diet and physical activity for the remission of type 2 diabetes: a paradigm shift | 2019 | not clinical trial |
| 240 | Davis | An Intensive Lifestyle Intervention to Treat Type 2 Diabetes in the Republic of the Marshall Islands: Protocol for a Randomized Controlled Trial | 2019 | not clinical trial |
| 241 | de Sousa | Biomarkers of insulin action during single soccer sessions before and after a 12-week training period in type 2 diabetes patients on a caloric-restricted diet | 2019 | not elderly patients |
| 242 | Elrefaey | Acute aerobic exercise induced irisin release in type 2 diabetic patients: Randomized clinical trial | 2019 | not elderly patients |
| 243 | Faroqi | STRONG-D: Strength training regimen for normal weight diabetics: Rationale and design | 2019 | not clinical trial |
| 244 | Fatimathu | Lifestyle practices and glycemic control among type 2 diabetic patients in north kerala | 2019 | not elderly patients |
| 245 | Gulsin | Rationale, design and study protocol of the randomised controlled trial: Diabetes Interventional Assessment of Slimming or Training tO Lessen Inconspicuous Cardiovascular Dysfunction (the DIASTOLIC study) | 2019 | not clinical trial |
| 246 | Hale | Protocol for a randomised controlled trial to evaluate the effectiveness of the diabetes community exercise and education programme (DCEP) for long-term management of diabetes | 2019 | not clinical trial |
| 247 | Ildarabadi | Effects of face-to-face and online training on self-care of middle-aged and elderly people with type 2 diabetes: A comparative study | 2019 | not elderly patients |
| 248 | Kitic | Study protocol for a multicentre, controlled non-randomised trial: benefits of exercise physiology services for type 2 diabetes (BEST) | 2019 | not clinical trial |
| 249 | Krankel | Exercise training to reduce cardiovascular risk in patients with metabolic syndrome and type 2 diabetes mellitus: How does it work? | 2019 | not clinical trial |
| 250 | Lucertini | Discontinuously supervised aerobic training vs. physical activity promotion in the self-management of type 2 diabetes in older Italian patients: design and methods of the 'TRIPL-A' randomized controlled trial | 2019 | not clinical trial |
| 251 | Magalhaes | Effects of combined training with different intensities on vascular health in patients with type 2 diabetes: a 1-year randomized controlled trial | 2019 | not elderly patients |
| 252 | Mendes | High-Intensity Interval Training Versus Moderate-Intensity Continuous Training in Middle-Aged and Older Patients with Type 2 Diabetes: A Randomized Controlled Crossover Trial of the Acute Effects of Treadmill Walking on Glycemic Control | 2019 | not elderly patients |
| 253 | Mey | Skeletal muscle Nur77 and NOR1 insulin responsiveness is blunted in obesity and type 2 diabetes but improved after exercise training | 2019 | not elderly patients |
| 254 | Motkova | MULTIDISCIPLINARY LIFESTYLE MANAGEMENT APPROACH IN PATIENTS WITH TYPE 2 DIABETES MELLITUS IN REAL CLINICAL PRACTICE. RESULTS OF APPLICATION "LIFE IS EASY" PROGRAMME IN RUSSIA | 2019 | not elderly patients |
| 255 | Olioso | Effects of Aerobic and Resistance Training on Circulating Micro-RNA Expression Profile in Subjects With Type 2 Diabetes | 2019 | not elderly patients |
| 256 | Piche | Benefits of 1-Year Lifestyle Modification Program on Exercise Capacity and Diastolic Function Among Coronary Artery Disease Men With and Without Type 2 Diabetes | 2019 | not elderly patients |
| 257 | Stubbs | Randomized Controlled Trial of Physical Exercise in Diabetic Veterans With Length-Dependent Distal Symmetric Polyneuropathy | 2019 | not elderly patients |
| 258 | Suryanegara | High intensity interval training protects the heart during increased metabolic demand in patients with type 2 diabetes: a randomised controlled trial | 2019 | not elderly patients |
| 259 | Szilagyi | Sports therapy and recreation exercise program in type 2 diabetes: randomized controlled trial, 3-month follow-up | 2019 | not elderly patients |
| 260 | Theresa | The effects of poco-poco dance on the executive functions of type 2 diabetes mellitus patients with mild cognitive impairment achieved through improvements in neuronal functions and plasticity | 2019 | not elderly patients |
| 261 | Viana | Prescribing high-intensity interval exercise by RPE in individuals with type 2 diabetes: metabolic and hemodynamic responses | 2019 | not elderly patients |
| 262 | Wang | Health literacy and exercise-focused interventions on clinical measurements in Chinese diabetes patients: A cluster randomized controlled trial | 2019 | not elderly patients |
| 263 | Wilson | HIIT Improves Left Ventricular Exercise Response in Adults with Type 2 Diabetes | 2019 | not elderly patients |
| 264 | Zaidi | Effects of exercise training on inflammasome-related mediators and their associations to glucometabolic variables in patients with combined coronary artery disease and type 2 diabetes mellitus: Sub-study of a randomized control trial | 2019 | not elderly patients |
| 265 | Zurlo | One-year caloric restriction and 12-week exercise training intervention in obese adults with type 2 diabetes: emphasis on metabolic control and resting metabolic rate | 2019 | not elderly patients |
| 266 | Arefirad | Effects of interval training on cardio metabolic risk factors and nitric oxide in type 2 diabetes patients: a randomized controlled trial | 2020 | not elderly patients |
| 267 | Benham | Significant Dose-Response between Exercise Adherence and Hemoglobin A1c Change | 2020 | not elderly patients |
| 268 | Borhade | Diabetes mellitus and exercise | 2020 | not clinical trial |
| 269 | Cox | Glycemic excursion minimization in the management of type 2 diabetes: a novel intervention tested in a randomized clinical trial | 2020 | not elderly patients |
| 270 | Furlano | Feasibility of a 26-Week Exercise Program to Improve Brain Health in Older Adults at Risk for Type 2 Diabetes: A Pilot Study | 2020 | not elderly patients |
| 271 | Ghardashi | Improved carotid intima-media thickness-induced high-intensity interval training associated with decreased serum levels of Dkk-1 and sclerostin in type 2 diabetes | 2020 | not elderly patients |
| 272 | Gholami | Cycle Training improves vascular function and neuropathic symptoms in patients with type 2 diabetes and peripheral neuropathy: A randomized controlled trial | 2020 | not elderly patients |
| 273 | Gupta | Effectiveness of Yoga-based Exercise Program Compared to Usual Care, in Improving HbA1c in Individuals with Type 2 Diabetes: A Randomized Control Trial | 2020 | not elderly patients |
| 274 | Hegde | Effects of Yoga Versus Sham Yoga on Oxidative Stress, Glycemic Status, and Anthropometry in Type 2 Diabetes Mellitus: A Single-Blinded Randomized Pilot Study | 2020 | not elderly patients |
| 275 | Iwai | Usefulness of Exercise for Home Blood Pressure Control in People with Diabetes: A Study Protocol for a Crossover Randomized Controlled Trial | 2020 | not clinical trial |
| 276 | Kaplan | The Effect of the Transtheoretical Model Based Walking Exercise Training and Follow-Up on Improving Exercise Behavior and Metabolic Control in Patients with Type 2 Diabetes | 2020 | not elderly patients |
| 277 | Leite | Impact of a supervised multicomponent physical exercise program on cognitive functions in patients with type 2 diabetes | 2020 | no outcome |
| 278 | Li | Effects of fitness qigong and tai chi on middle-aged and elderly patients with type 2 diabetes mellitus | 2020 | not elderly patients |
| 279 | Machado | Acute and chronic effects of muscle power training on blood pressure in elderly patients with type 2 diabetes mellitus | 2020 | without control group |
| 280 | ranasinghe | Glycemic and cardiometabolic effects of exercise in South Asian Sri Lankans with type 2 diabetes mellitus: A randomized controlled trial Sri Lanka diabetes aerobic and resistance training study (SL-DARTS) | 2020 | not elderly patients |
| 281 | Seyam | Effect of Walking on Sand with Dietary Intervention in OverweightType 2 DiabetesMellitusPatients: A Randomized Controlled Trial | 2020 | not elderly patients |
| 282 | Sorensen | Higher levels of daily physical activity are associated with better skin microvascular function in type 2 diabetes—The Maastricht Study | 2020 | not elderly patients |
| 283 | Verboven | Impact of Exercise-Nutritional State Interactions in Patients with Type 2 Diabetes | 2020 | without control group |
| 284 | Wang | Study on the effect of lifestyle intervention on metabolic indexes of rural patients with type 2 diabetes mellitus | 2020 | without control group |
| 285 | Wasenius | Low-cost exercise interventions improve long-term cardiometabolic health independently of a family history of type 2 diabetes: a randomized parallel group trial | 2020 | not elderly patients |
| 286 | Way | The effect of low-volume high-intensity interval training on cardiovascular health outcomes in type 2 diabetes: A randomised controlled trial | 2020 | not elderly patients |
| 287 | Wei | Health literacy and exercise interventions on clinical outcomes in Chinese patients with diabetes: a propensity score-matched comparison | 2020 | not elderly patients |
| 288 | Whipple | Influence of Changes in Sedentary Time on Outcomes of Supervised Exercise Therapy in Individuals with Comorbid Peripheral Artery Disease and Type 2 Diabetes | 2020 | not elderly patients |
| 289 | Yang | The impact of exercise on serum irisin, osteocalcin, and adiponectin levels and on glycolipid metabolism in patients with type 2 diabetes | 2020 | not elderly patients |
| 290 | Bilak | Cardiovascular and systemic determinants of exercise capacity in people with type 2 diabetes mellitus | 2020 | not clinical trial |
| 291 | Bilger | Process- and Outcome-Based Financial Incentives to Improve Self-Management and Glycemic Control in People with Type 2 Diabetes in Singapore: A Randomized Controlled Trial | 2020 | not elderly patients |
| 292 | MacDonald | One-year intensive lifestyle intervention and improvements in health-related quality of life and mental health in persons with type 2 diabetes: A secondary analysis of the U-TURN randomized controlled trial | 2020 | not elderly patients |
| 293 | Barragan | Effects of exercise on glycosylated hemoglobin type 2 diabetic patients from the West of Mexico | 2011 | not available |
| 294 | Wang | Skeletal muscle and subcutaneous adipose tissue adaptations to power training in older adults with type 2 diabetes: a double blind, randomized sham-exercise controlled trial | 2011 | not available |
| 295 | Subramanian | Physio ball exercises on obesity and type II diabetes | 2014 | not available |
| 296 | Steurer | Endurance training and walking improve blood glucose metabolism in type 2 diabetes mellitus | 2015 | not available |
| 297 | Moosavi | The effect of regular aerobic exercise on plasma levels of 25-hydroxy Vitamin D and insulin resistance in hypertensive postmenopausal women with type 2 diabetes | 2016 | not available |
| 298 | Ahmed | Efficacy of high intensity interval training on endothelial function in diabetics with peripheral arterial insufficiency | 2019 | not available |

**Material suplementario 3.** Características de los estudios incluidos en la revisión sistemática (n=7).

| **Estudio** | **País** | **Diseño** | **Principal criterio de inclusión** | **Principal criterio de exclusión** | **Tamaño analizado de muestra (I/C)** | **Ejercicio intervención** | **Tiempo de intervención  (h/s; seguimiento)** | **Comparador** | **Efecto adverso** | **Resultado primario** | **Conclusión** | **Fuente de finamiento** |
| --- | --- | --- | --- | --- | --- | --- | --- | --- | --- | --- | --- | --- |
| Madden, 2010^34^ | Canadá | Estudio randomizado simple ciego | Mayores de 65 años, DT2 durante al menos 5 años, ECG normal, prueba de esfuerzo máximo en cinta rodante normal | Antecedentes de angina, infarto de miocardio, accidente cerebrovascular, enfermedad pulmonar crónica | 19/20 | Caminadora y bicicleta ergometrica | 3h/s; 12 semanas | Ejercicio no aeróbico core | No reportado | Cambio en la función barorrefleja | El ejercicio aeróbico puede revertir las deficiencias funcionales de la función barorrefleja arterial | “Canadian Institutes of Health Research” |
| Sung, 2012^36^ | Korea | Estudio randomizado etiqueta abierta | Mayor de 65 años con DT2, obtuvo más de 24 puntos en el Mini-Examen del Estado Mental | Participantes con consumo habitual de alcohol o enfermedad hepática | 22/18 | Caminar | 2.5h/s; 24 semanas. | Cuidado de salud standard | No reportado | Diet control and the management of complications | El ejercicio aeróbico aumenta la actividad diaria y el metabolismo de la glucosa | “National Research Foundation” Grant financiado por el gobierno de Korea |
| Madden,2013^35^ | Canadá | Estudio randomizado simple ciego | Mayor de 65 años con DT2 durante al menos 5 años, ECG normal, sedentario al inicio del estudio | Antecedentes de angina, infarto de miocardio, accidente cerebrovascular, enfermedad pulmonar crónica, tabaquismo en los últimos 5 años | 20/20 | Caminadora y bicicleta ergometrica | 3h/s; 12 semanas | Pelota de ejercicio | Intervención: Respuesta sincopal con masaje del seno carotideo: 1 (5%) | Atenuación de la respuesta vasodepresiva | La hipersensibilidad vasodepresiva del seno carotídeo es resistente al ejercicio aeróbico | “Canadian  Institutes of Health Research” y “Academic Enhancement  Fund” |
| Parra-Sánchez,2015^37^ | España | Estudio randomizado simple ciego | Edad entre 65-80 años, DT2 | Incapacidad para reconocer, no ser sedentario, antecedentes de retinopatía, nefropatía avanzada, insuficiencia cardiaca congestiva | 47/41 | Caminar | 2h/s;12 semanas | No Ejercicio | Intervención: ictus: 1 (2%), esguince: 1 (2%)  Control: ictus: 1 (2%) | Cambios en hemoglobina glicosilada | La hipersensibilidad vasodepresiva del seno carotídeo es resistente | Fundación para la Formación y la  Investigación de los Profesionales de la Salud de Extremadura (FundeSalud). |
| Askari, 201838 | Irán | Estudio randomizado etiqueta abierta | Mayores de 60 años, T2D durante al menos 1 año, falta de discapacidades cognitivas y de movimiento. | Ausencia de >2 sesiones en el programa de entrenamiento | 54/54 | Caminar y trotar | 1 h/s, 12 semanas | No detalles | No reportado | Cambio en los índices metabólicos | El ejercicio aeróbico mejora los índices metabólicos | Ninguna |
| Sijie Tan, 2018^39^ | China | Estudio randomizado etiqueta abierta | Mujeres con DT2 entre 6-10 años, edad entre 60-69 años | Incapacidad para participar en el ejercicio, antecedentes de enfermedad cardíaca, presión arterial superior a 160/95 mmhg, enfermedades pulmonares, | 16/15 | Caminar o correr | 3 h/s; 12 semanas | Niveles habituales de ejercicio físico | No reportado | Cambio en la composición corporal, glucosa en sangre, perfil de lípidos, adipocina sérica y resistencia a la insulina | El ejercicio aeróbico mejora la composición corporal, la glucosa en sangre y la resistencia a la insulina | “National Physical Fitness Promotion Plan of General Administration of Sport of  China” y “The Tianjin National Science Program” |
| Hagag, 2020^40^ | Egipto | Randomized open-label study | Edad entre 60 y 70 años, IMC entre 25 y 34,9, DT2 entre 2 y 7 años | Antecedentes de dislipidemia, hipertensión, complicaciones de DT2 | 20/20 | Caminadora | 2.25h/s; 12 semanas | Cuidado de salud standard | No reportado | Cambio en la defensa antioxidante y factores de riesgo cardiometabólicos | El ejercicio aeróbico es efectivo sobre los riesgos cardiometabólicos, los marcadores de estrés oxidativo y el estado antioxidante | Ninguna |
| I: Intervención, C: comparador, h: horas, s: Semanas, T2D: diabetes tipo 2, ECG: Ecocardiografia | | | | | | | | | | | |  |

**Material suplementario 4.** Riesgo de sesgo de los estudios incluidos basado en la herramienta de Cochrane de riesgo de sesgo

| **Estudio** | **Proceso de aleatorización** | **Desviación de las intervenciones previstas** | **Falta de datos sobre el resultado** | **Medición de los resultados** | **Selección del resultado informado** | **General** |
| --- | --- | --- | --- | --- | --- | --- |
| **Madden (2010)** |  |  |  |  |  |  |
| **Sung (2012)** |  |  |  |  |  |  |
| **Madden (2013)** |  |  |  |  |  |  |
| **Parra-Sanchez (2015)** |  |  |  |  |  |  |
| **Askari (2018)** |  |  |  |  |  |  |
| **Sijie (2018)** |  |  |  |  |  |  |
| **Hagag (2020)** |  |  |  |  |  |  |

**Material suplementario 5.** Análisis de sensibilidad

| **Subgrupo** | **Resultado**  **DME (95%IC)** | **Estudio excluido** | **Resultados después de la exclusion de cada estudio DME (95% IC)** |
| --- | --- | --- | --- |
|  |  |  |  |
| **Glucosa en ayunas** | -1.76 [-2.78; -0.74] |  |  |
|  |  | Madden(2010) | -1.47 [-2.39; -0.47] |
|  |  | Sung(2012) | -1.96 [-3.33; -0.58] |
|  |  | Madden(2013) | -1.49 [-2.51; -0.47] |
|  |  | Askari(2018) | -2.11 [-3.09; -1.13] |
|  |  | Sijie(2018) | -1.82 [-3.09; -0.55] |
| **Triglicéridos** | -0.34 [-0.67; -0.01] |  |  |
|  |  | Sung(2012) | -0.25 [-0.57; 0.08] |
|  |  | Parra - Sanchez(2015) | -0.44 [-0.71; -0.17] |
|  |  | Askari(2018) | -0.40 [-0.87; 0.07] |
|  |  | Sijie(2018) | -0.26 [-0.59; 0.07] |
|  |  | Hagag(2020) | -0.36 [-0.76; 0.05] |
| **HDL** | 0.41 [0.11; 0.72] |  |  |
|  |  | Sung(2012) | 0.39 [0.02; 0.77] |
|  |  | Parra - Sanchez(2015) | 0.59 [0.32; 0.86] |
|  |  | Askari(2018) | 0.24 [-0.03; 0.51] |
|  |  | Sijie(2018) | 0.44 [0.07; 0.80] |
|  |  | Hagag(2020) | 0.41 [0.03; 0.79] |
| **Presión arterial sistólica** | -1.67 [-2.74; -0.61] |  |  |
|  |  | Madden(2010) | -1.41 [-2.55; -0.26] |
|  |  | Madden(2013) | -1.32 [-2.35; -0.29] |
|  |  | Parra - Sanchez(2015) | -2.08 [-3.19; -0.97] |
|  |  | Hagag(2020) | -1.91 [-3.49; -0.33] |
| **Presión arterial diastólica** | -1.47 [-2.34; -0.60] |  |  |
|  |  | Madden(2010) | -1.50 [-2.68; -0.31] |
|  |  | Madden(2013) | -1.00 [-1.52; -0.48] |
|  |  | Parra - Sanchez(2015) | -1.80 [-2.79; -0.80] |
|  |  | Hagag(2020) | -1.62 [-2.87; -0.37] |
|  |  |  |  |
| **Hemoglobina glicosilada** | -0.63 [-0.87; -0.39] |  |  |
|  |  | Sung(2012) | -0.60 [-0.88; -0.32] |
|  |  | Parra - Sanchez(2015) | -0.78 [-1.08; -0.48] |
|  |  | Askari(2018) | -0.60 [-0.95; -0.26] |
|  |  | Hagag(2020) | -0.60 [-0.87; -0.32] |
| DME: Diferencia de medias estandarizada IC: Intervalo de confianza  HDL: high density lipoprotein | | | |

| **Material suplementario 6.** Análisis por subgrupos | | | | | |
| --- | --- | --- | --- | --- | --- |
| **Subgrupo** | **n** | **Resultado** | | **Prueba de heterogeneidad** | |
|  |  | **DME (95% CI)** | **P valor** | **I²** | **P valor** |
| **Glucosa en ayunas** |  |  |  |  |  |
| **Intensidad de ejercicio** |  |  |  |  |  |
| Alta o vigoroso | 2 | -2.05 [-4.05; -0.06] | 0.04 | 92% | <0.01 |
| Bajo o moderado | 1 | -1.57 [-2.39; -0.75] | <0.01 |  |  |
| No reportado | 2 | -1.62 [-3.96; 0.72] | 0.17 | 96% | <0.01 |
| **Supervisión de ejercicio** |  |  |  |  |  |
| Si | 3 | -2.48 [-3.44; -1.53] | <0.01 | 71% | 0.03 |
| No | 2 | -0.70 [-1.27; -0.12] | 0.02 | 57% | 0.13 |
| **Duración total en horas** |  |  |  |  |  |
| >30 | 4 | -2.11 [-3.09; -1.13] | <0.01 | 82% | <0.01 |
| ≤30 | 1 | -0.46 [-0.85; -0.08] | 0.02 |  |  |
| **Triglicéridos** |  |  |  |  |  |
| **Intensidad de ejercicio** |  |  |  |  |  |
| Alta o vigoroso | 1 | -0.78 [-1.43; -0.13] | 0.02 |  |  |
| Bajo o moderado | 2 | -0.54 [-1.02; -0.07] | 0.03 | 0% | 0.37 |
| No reportado | 2 | -0.09 [-0.44; 0.26] | 0.60 | 39% | 0.20 |
| **Supervisión de ejercicio** |  |  |  |  |  |
| Si | 3 | -0.28 [-0.80; 0.23] | 0.28 | 59% | 0.09 |
| No | 2 | -0.45 [-0.94; 0.03] | 0.07 | 44% | 0.18 |
| **Duración total en horas** |  |  |  |  |  |
| >30 | 2 | -0.79 [-1.28; -0.30] | <0.01 | 0% | 0.96 |
| ≤30 | 3 | -0.14 [-0.40; 0.13] | 0.31 | 9% | 0.33 |
| **HDL** |  |  |  |  |  |
| **Intensidad de ejercicio** |  |  |  |  |  |
| Alta o vigoroso | 1 | 0.52 [-0.12; 1.15] | 0.11 |  |  |
| Bajo o moderado | 2 | 0.37 [-0.10; 0.84] | 0.13 | 0% | 0.71 |
| No reportado | 2 | 0.41 [-0.29; 1.12] | 0.25 | 85% | 0.01 |
| **Supervisión de ejercicio** |  |  |  |  |  |
| Si | 3 | 0.18 [-0.12; 0.48] | 0.24 | 0% | 0.56 |
| No | 2 | 0.70 [0.37; 1.03] | <0.01 | 0% | 0.5 |
| **Duración total en horas** |  |  |  |  |  |
| >30 | 2 | 0.40 [-0.07; 0.88] | 0.09 | 0% | 0.60 |
| ≤30 | 3 | 0.42 [-0.06; 0.90] | 0.08 | 69% | 0.04 |
| **Presión arterial sistólica** |  |  |  |  |  |
| **Intensidad de ejercicio** |  |  |  |  |  |
| Alta o vigoroso | 1 | -2.50 [-3.34; -1.65] | <0.01 |  |  |
| Bajo o moderado | 1 | -1.06 [-1.73; -0.39] | <0.01 |  |  |
| No reportado | 2 | -1.63 [-3.79; 0.53] | 0.14 | 95% | <0.01 |
| **Supervisión de ejercicio** |  |  |  |  |  |
| Si | 4 | -1.67 [-2.24; -0.61] | <0.01 | 90% | <0.01 |
| No | 0 |  | -- |  |  |
| **Duración total en horas** |  |  |  |  |  |
| >30 | 2 | -2.48 [-3.24; -2.01] | <0.01 | 0% | 0.66 |
| ≤30 | 2 | -0.74 [-1.20; -0.27] | <0.01 | 36% | 0.21 |
| **Presión arterial diastólica** |  |  |  |  |  |
| **Intensidad de ejercicio** |  |  |  |  |  |
| Alta o vigoroso | 1 | -1.47 [-2.18; -0.76] | <0.01 |  |  |
| Bajo o moderado | 2 | -1.74 [-4.01; 0.53] | 0.13 | 95% | <0.01 |
| No reportado | 1 | -1.11 [-1.78; -0.44] | <0.01 |  |  |
| **Supervisión de ejercicio** |  |  |  |  |  |
| Si | 4 | -1.47 [-2.34; -0.60] | <0.01 | 86% | <0.01 |
| No | 0 |  |  |  |  |
| **Duración total en horas** |  |  |  |  |  |
| >30 | 2 | -2.17 [-3.61; -0.74] | <0.01 | 84% | 0.01 |
| ≤30 | 2 | -0.79 [-1.25; -0.33] | <0.01 | 34% | 0.22 |
| **Hemoglobina glicosilada** |  |  |  |  |  |
| **Intensidad de ejercicio** |  |  |  |  |  |
| Alta o vigoroso | 1 | -0.85 [-1.50; -0.20] | 0.01 |  |  |
| Bajo o moderado | 1 | -0.86 [-1.52; -0.21] | <0.01 |  |  |
| No reportado | 2 | -0.55 [-0.89; -0.20] | <0.01 | 35% | 0.22 |
| **Supervisión de ejercicio** |  |  |  |  |  |
| Si | 2 | -0.55 [-1.01; -0.08] | 0.02 | 38% | 0.20 |
| No | 2 | -0.76 [-1.09; -0.42] | <0.01 | 0% | 0.74 |
| **Duración total en horas** |  |  |  |  |  |
| >30 | 1 | -0.85 [-1.50; -0.20] | 0.01 |  |  |
| ≤30 | 3 | -0.60 [-0.88; -0.32] | <0.01 | 13% | 0.32 |
| n: numero de estudios, DME: Diferencia de medias estandarizada IC: Intervalo de confianza   1. La intensidad del ejercicio se clasifico de acuerdo al studio por Norton^12^ 2. El estudio se considera supervisado si fue supervisado por algún profesional de la salud, entrenador personal de manera presencial o virtual 3. El punto de corte de 30 horas para la duracion total en horas de ejercicio aerobico se basó en el studio por Shiroma E. et al^30^ 4. La deficinión de ejrcicio aeróbico se basó en la “Physical Activity Guidelines for Americans”^22^ | | | | | |

| **Material suplementario 7.** PRISMA checklist | | | |
| --- | --- | --- | --- |
| **Section and Topic** | **Item #** | **Checklist item** | **Location where item is reported** |
| **TITLE** | | |  |
| Title | 1 | Identify the report as a systematic review. | Page 1 |
| **ABSTRACT** | | |  |
| Abstract | 2 | See the PRISMA 2020 for Abstracts checklist. | Page 3 |
| **INTRODUCTION** | | |  |
| Rationale | 3 | Describe the rationale for the review in the context of existing knowledge. | Page 4 |
| Objectives | 4 | Provide an explicit statement of the objective(s) or question(s) the review addresses. | Page 4 |
| **METHODS** | | |  |
| Eligibility criteria | 5 | Specify the inclusion and exclusion criteria for the review and how studies were grouped for the syntheses. | Page 5 |
| Information sources | 6 | Specify all databases, registers, websites, organisations, reference lists and other sources searched or consulted to identify studies. Specify the date when each source was last searched or consulted. | Page 6 |
| Search strategy | 7 | Present the full search strategies for all databases, registers and websites, including any filters and limits used. | Supplementary Material 1 |
| Selection process | 8 | Specify the methods used to decide whether a study met the inclusion criteria of the review, including how many reviewers screened each record and each report retrieved, whether they worked independently, and if applicable, details of automation tools used in the process. | Page 6 |
| Data collection process | 9 | Specify the methods used to collect data from reports, including how many reviewers collected data from each report, whether they worked independently, any processes for obtaining or confirming data from study investigators, and if applicable, details of automation tools used in the process. | Page 6 |
| Data items | 10a | List and define all outcomes for which data were sought. Specify whether all results that were compatible with each outcome domain in each study were sought (e.g. for all measures, time points, analyses), and if not, the methods used to decide which results to collect. | Page 6 |
|  | 10b | List and define all other variables for which data were sought (e.g. participant and intervention characteristics, funding sources). Describe any assumptions made about any missing or unclear information. | Page 5 |
| Study risk of bias assessment | 11 | Specify the methods used to assess risk of bias in the included studies, including details of the tool(s) used, how many reviewers assessed each study and whether they worked independently, and if applicable, details of automation tools used in the process. | Page 7 |
| Effect measures | 12 | Specify for each outcome the effect measure(s) (e.g. risk ratio, mean difference) used in the synthesis or presentation of results. | Pages 7 - 8 |
| Synthesis methods | 13a | Describe the processes used to decide which studies were eligible for each synthesis (e.g. tabulating the study intervention characteristics and comparing against the planned groups for each synthesis (item #5)). | Page 6 |
|  | 13b | Describe any methods required to prepare the data for presentation or synthesis, such as handling of missing summary statistics, or data conversions. | Page 7 |
|  | 13c | Describe any methods used to tabulate or visually display results of individual studies and syntheses. | Page 8 |
|  | 13d | Describe any methods used to synthesize results and provide a rationale for the choice(s). If meta-analysis was performed, describe the model(s), method(s) to identify the presence and extent of statistical heterogeneity, and software package(s) used. | Page 8 |
|  | 13e | Describe any methods used to explore possible causes of heterogeneity among study results (e.g. subgroup analysis, meta-regression). | Pages 7 - 8 |
|  | 13f | Describe any sensitivity analyses conducted to assess robustness of the synthesized results. | Page 8 |
| Reporting bias assessment | 14 | Describe any methods used to assess risk of bias due to missing results in a synthesis (arising from reporting biases). | Page 8 |
| Certainty assessment | 15 | Describe any methods used to assess certainty (or confidence) in the body of evidence for an outcome. | Page 8 |
| **RESULTS** | | |  |
| Study selection | 16a | Describe the results of the search and selection process, from the number of records identified in the search to the number of studies included in the review, ideally using a flow diagram. | Figure 1 |
|  | 16b | Cite studies that might appear to meet the inclusion criteria, but which were excluded, and explain why they were excluded. | Supplementary Material 2 |
| Study characteristics | 17 | Cite each included study and present its characteristics. | Supplementary Material 3 |
| Risk of bias in studies | 18 | Present assessments of risk of bias for each included study. | Supplementary Material 4 |
| Results of individual studies | 19 | For all outcomes, present, for each study: (a) summary statistics for each group (where appropriate) and (b) an effect estimate and its precision (e.g. confidence/credible interval), ideally using structured tables or plots. | Figure 2 - 4 |
| Results of syntheses | 20a | For each synthesis, briefly summarise the characteristics and risk of bias among contributing studies. | Page 9 |
|  | 20b | Present results of all statistical syntheses conducted. If meta-analysis was done, present for each the summary estimate and its precision (e.g. confidence/credible interval) and measures of statistical heterogeneity. If comparing groups, describe the direction of the effect. | Pages 9 - 11 |
|  | 20c | Present results of all investigations of possible causes of heterogeneity among study results. | Pages 9 - 11 |
|  | 20d | Present results of all sensitivity analyses conducted to assess the robustness of the synthesized results. | Supplementary Material 4 |
| Reporting biases | 21 | Present assessments of risk of bias due to missing results (arising from reporting biases) for each synthesis assessed. | Supplementary Material 4 |
| Certainty of evidence | 22 | Present assessments of certainty (or confidence) in the body of evidence for each outcome assessed. | Table 1 |
| **DISCUSSION** | | |  |
| Discussion | 23a | Provide a general interpretation of the results in the context of other evidence. | Pages 11 - 14 |
|  | 23b | Discuss any limitations of the evidence included in the review. | Page 15 |
|  | 23c | Discuss any limitations of the review processes used. | Page 15 |
|  | 23d | Discuss implications of the results for practice, policy, and future research. | Page 15 |
| **OTHER INFORMATION** | | |  |
| Registration and protocol | 24a | Provide registration information for the review, including register name and registration number, or state that the review was not registered. | Page 5 |
|  | 24b | Indicate where the review protocol can be accessed, or state that a protocol was not prepared. | Page 5 |
|  | 24c | Describe and explain any amendments to information provided at registration or in the protocol. | Page 5 |
| Support | 25 | Describe sources of financial or non-financial support for the review, and the role of the funders or sponsors in the review. | Page 16 |
| Competing interests | 26 | Declare any competing interests of review authors. | Page 16 |
| Availability of data, code and other materials | 27 | Report which of the following are publicly available and where they can be found: template data collection forms; data extracted from included studies; data used for all analyses; analytic code; any other materials used in the review. | Supplementary material 3 |
