# Supplementary material for: Effects of aerobic exercise on components of the metabolic syndrome in older adults with type 2 diabetes mellitus: systematic review and meta-analysis
Source: Rev Peru Med Exp Salud Publica. 2024 Jun 11;41(2):146–55. doi: 10.17843/rpmesp.2024.412.12751 (PMC11300696; doi:10.17843/rpmesp.2024.412.12751)
Supplement: Translation

# Translation: Efectos del ejercicio aeróbico sobre los componentes del síndrome metabólico en adultos mayores con diabetes *mellitus* tipo 2: revisión sistemática y metaanálisis

**Authors:** Mercedes Miranda-Tueros, Joshua Ramirez-Peña, Miguel Cabanillas-Lazo, José Luis Paz-Ibarra, Isabel Pinedo-Torres

**Affiliations:** 1 Universidad Científica del Sur, Facultad de Ciencias de la Salud, Lima, Perú.; 2 Red de Eficacia Clínica y Sanitaria, REDECS, Lima, Perú.; 3 Universidad de Huánuco, Huánuco, Perú.; 4 Facultad de Medicina, Universidad Nacional Mayor de San Marcos, Lima, Perú.; 5 Hospital Nacional Edgardo Rebagliati Martins, Lima, Perú.; 6 Grupo de Investigación NEURONECS: Neurociencia, efectividad clínica y salud pública, Universidad Científica del Sur, Lima, Perú.

**Keywords:** Ejercicio Físico, Diabetes Mellitus, Anciano, Revisión Sistemática, Síndrome Metabólico

## Abstract

**Objetivo.:**

Determinar los efectos del ejercicio aeróbico sobre los componentes del síndrome metabólico en pacientes adultos mayores diabéticos mediante una revisión sistemática con meta-análisis.

**Materiales y métodos.:**

Utilizamos las bases de datos PubMed/Medline, Scopus, Cochrane library, Web of Science y el buscador Google Scholar. Se seleccionaron los ensayos controlados aleatorizados (ECA) según los criterios de inclusión. Dos revisores determinaron de forma independiente si los estudios cumplían con los criterios de inclusión, extrajeron los datos y utilizaron la herramienta Cochrane de riesgo de sesgo (RoB 2). Los análisis cuantitativos se realizaron en el programa R v 4.0.5, utilizando efectos aleatorios.

**Resultados.:**

Las búsquedas identificaron 8697 estudios, de los que siete ECA se incluyeron en la síntesis cualitativa. Se evaluó que la mayoría de los estudios tenían un RoB alto o bajo en al menos tres dominios. El metaanálisis mostró que el ejercicio aeróbico fue eficaz para mejorar los niveles de glucosa (diferencia de medias estandarizada [DME]: -1,04; intervalo de confianza al 95% [IC 95%] -1,27; -0,81), presión arterial sistólica (DME: -0,79; IC 95%: -1,02; -0,56), presión arterial diastólica (DME: -0,75; IC 95%: -0,98; -0,52), hemoglobina glucosilada (DME: -0,57; IC 95%: -0,77; -0,37), HDL (DME: 0,35; IC 95%: 0,15; 0,55), triglicéridos (DME: -0,26; IC 95%: -0,47; -0,06). No se informaron efectos adversos significativos. El nivel de certeza de los resultados fue baja para la glucosa en ayunas, moderado para la presión arterial sistólica y diastólica, y muy bajo para los demás resultados, además de pocos efectos adversos. Sin embargo, estos resultados deben ser interpretados con cautela debido al uso de marcadores subrogados.

**Conclusiones.:**

El ejercicio aeróbico demostró tener una mejoría significativa en los componentes del síndrome metabólico en adultos mayores diabéticos y no se reportaron efectos adversos importantes. Sin embargo, recomendamos más ECA que tengan un mayor tiempo de intervención para establecer el impacto sobre los síntomas y las complicaciones.

## INTRODUCCIÓN

Se estima que para el año 2050, la población de adultos mayores será el doble que la reportada en la proyección de la población del año 2019, lo que significa que una de cada seis personas en el mundo tendrá 65 años o más ^1^. Además, el síndrome metabólico, y su complicación, la diabetes mellitus es muy frecuente en esta población ^2^^,^^3^. Este síndrome incluye la alteración de tres o más de los siguientes signos: presión arterial sistólica, diastólica, colesterol asociado a lipoproteínas de alta densidad, triglicéridos, y la glucosa en ayuna; sobre este último la valoración de la hemoglobina glicosilada es más útil en personas con diabetes debido a su alto valor clínico y buena precisión diagnostica en complicaciones de la hiperglicemia ^4^. Inclusive ha sido utilizado en conjunto con la glucosa en ayunas como método de *screening* para el síndrome metabólico y su inclusión se ha considerado en los criterios del síndrome metabólico ^5^^,^^6^. Cabe resaltar que la presencia estas dos condiciones (diabetes y síndrome metabólico) aumentan el riesgo de complicaciones cardiovasculares fatales a corto plazo, como isquemias cardiacas o enfermedad cerebrovascular ^7^^,^^8^.

Actualmente, las guías disponibles recomiendan la realización de actividad física junto con la dieta y medicación para mejorar los parámetros glucémicos y lipídicos en pacientes con diabetes ^9^^,^^10^. Dentro del entrenamiento físico, existen varios tipos de ejercicios de utilidad demostrada, como el ejercicio aeróbico, de resistencia y de alta intensidad ^11^^,^^12^. El ejercicio aeróbico, definido como cualquier trabajo que desarrolle la aptitud cardiovascular y pulmonar, utiliza el oxígeno como sustrato metabólico ^13^ y es el más estudiado. Esto incluye entrenamientos de diferentes intensidades, desde caminar a paso ligero hasta trotar o nadar ^14^^,^^15^. Este tipo de ejercicio proporciona diversos beneficios, como una mejora en la sensibilidad a la insulina, aumento de la fibrinólisis, disminución de los triglicéridos y la presión arterial, todo lo cual mejora el pronóstico de un paciente con diabetes ^16^^-^^19^.

Sin embargo, el ejercicio físico no puede prescribirse sin restricciones. La población adulta mayor suele presentar limitación de movilidad, obesidad, discapacidad visual o enfermedad cardiovascular, y a la población geriátrica con diagnóstico de diabetes, se le suma un mayor riesgo de hipoglucemia y síndrome de fragilidad ^20^^-^^23^. Aunque las guías ^24^ recomiendan que los adultos mayores realicen al menos 150 minutos de actividad física aeróbica de intensidad moderada o 75 minutos de actividad física aeróbica de intensidad vigorosa por semana, los efectos potenciales del ejercicio aeróbico sobre los agentes metabólicos marcadores para el control adecuado de la diabetes en personas mayores y la seguridad de su prescripción no se conocen completamente ^25^. Es por esta razón, el objetivo de esta revisión sistemática con metaanálisis fue determinar los efectos del ejercicio aeróbico sobre los componentes del síndrome metabólico en pacientes adultos mayores con diabetes. Con el fin de proporcionar a los médicos generales y especialistas una visión completa de la evidencia científica sobre una posible opción de tratamiento no farmacológico para mejorar la salud de esta población.

**MENSAJES CLAVE**

Motivación para el estudio. La motivación de esta investigación surge ante la alta prevalencia mundial del síndrome metabólico y de diabetes mellitus. A pesar de su impacto, existe una brecha en el conocimiento sobre las intervenciones no farmacológicas en adultos mayores que puedan mejorar el perfil metabólico de estos pacientes.

Principales hallazgos. Los resultados mostraron una mejora significativa en los niveles de glucosa, presión arterial, hemoglobina glucosilada, HDL y triglicéridos después de la intervención con ejercicio aeróbico. Además, no se observaron efectos adversos significativos.

Implicancias en salud pública. El ejercicio físico es una estrategia asequible y disponible en todo el mundo. Mejora el perfil metabólico de pacientes adultos mayores con síndrome metabólico.

## MATERIALES Y MÉTODOS

Se llevó a cabo una revisión sistemática siguiendo las directrices del «Systematic Reviews and Meta-Analyses (PRISMA) 2020». El protocolo se registró en PROSPERO con el código de CRD42021250115.

### Estrategia de búsqueda

Se realizó una búsqueda sistemática en las bases de datos de PubMed/Medline, Scopus, Cochrane library, Web of Science y se usó el buscador Google Scholar para literatura gris. Se usaron términos MeSH y libres relacionados para «ejercicio», «diabetes *mellitus*». En vez de incluir truncadores se expandieron los términos relacionados a los principales, tales como «jogging», «treadmill», «swimming», «running» o «ambulation» (material suplementario).

### Criterios de selección

Se incluyeron ensayos clínicos aleatorizados (ECA) que cumplieron los siguientes criterios: a) pacientes con diabetes *mellitus* tipo 2 (DM2) con al menos uno de los componentes del síndrome metabólico, b) evaluación de adultos mayores (mayores de 60 años), c) disponibilidad de texto completo del artículo y d) intervención de cualquier tipo de ejercicio aeróbico durante un tiempo mínimo de 12 semanas en comparación con el estándar de atención o ninguna intervención. La definición de ejercicio aeróbico se consideró de acuerdo con las pautas de guía «Physical Activity Guidelines for Americans» realizadas por el Departamento de Salud y Servicios Humanos de los Estados Unidos la cual lo define como el movimiento de músculos largos del cuerpo de una manera rítmica por un periodo de tiempo sostenido ^14^. El ejercicio aeróbico incluye correr, caminar, usar bicicleta ergométrica y caminadora ^27^. También se tuvo en cuenta la intensidad del ejercicio según lo recomendado, considerando como sedentario (<1,6 METs, <40% HRmax, <20% HRR, <20% VO2 max, RPE (C): <8, RPE (C-R): <1), leve (1,6-3 METs, 40-55% HRmax, 20-40% HRR, 20-40% VO2 max, RPE (C): 8-10, RPE (C-R): 1-2), moderada (3-6 METs, 55-70% HRmax, 40-60% HRR, 40-60% VO2 max, RPE (C): 11-13, RPE (C-R): 3-4) y vigorosa (6-9 METs, 70-90% HRmax, 60-85% HRR, 60-85% VO2 max, RPE (C): 14-16, RPE (C-R): 5-6). Estas medidas de intensidad son abreviadas con la siguiente terminología, METs significa «equivalente metabólico», donde 1 MET equivale a 3,5 ml de O2/kg/min, % HRmax es la «frecuencia cardiaca máxima», %HRR alude a la «reserva de frecuencia cardíaca», que a su vez es la FC máxima - FC en reposo y %VO2max siendo el «consumo máximo de oxígeno». Por otro lado, las medidas subjetivas provienen de las escalas RPE de Borg del esfuerzo percibido, donde RPE-C, mide en una escala de categorías de 6-20 y RPE C-R, se miden en la escala de relación de categorías de 0-10 ^27^. No hubo restricción de la búsqueda por año. Se excluyó cualquier otro tipo de publicación (cartas al editor, reporte de casos, estudios observacionales, revisiones narrativas y sistemáticas).

### Selección de estudios y extracción de datos

Se importaron los resultados de la búsqueda electrónica al programa de gestión de referencias EndNote X9; seguido de esto, se eliminaron los registros duplicados de acuerdo con los procedimientos descritos por Bramer *et al.*^26^. Posteriormente, dos autores (MMT y JRP) examinaron de forma independiente los títulos y resúmenes de acuerdo con los criterios de selección. Se seleccionaron los estudios relevantes y se realizaron búsquedas de textos completos. Las discrepancias en las selecciones se resolvieron por consenso y, en última instancia, se consultó a un tercer autor (IPT). La lista completa de artículos excluidos en esta etapa del texto completo está disponible en el material suplementario 2. Dos autores (JRP y MCL) extrajeron de forma independiente los datos de cada artículo incluido utilizando un formulario estandarizado en Microsoft Excel. Un tercer autor (IPT) verificó la exactitud de los datos antes del análisis. Para los resultados dicotómicos, se extrajeron las frecuencias absolutas y relativas. Para los resultados continuos, se extrajeron las mediciones basales y de seguimiento, así como el cambio entre ellas.

### Análisis de resultados

Los resultados primarios fueron los componentes del síndrome metabólico según la Federación Internacional de Diabetes (FID) ^27^: glucosa en ayunas (mg/dl), presión arterial sistólica (PAS, mmHg), presión arterial diastólica (PAD, mmHg), lipoproteínas de alta densidad (HDL, mg/dl), triglicéridos (mg/dl) y circunferencia de la cintura (cm/pulgada). También se consideró la hemoglobina glicosilada (HbA1c, %) debido a la importancia clínica que representa, pese a no ser parte de los componentes del síndrome metabólico. Para interpretar el tamaño del efecto, se utilizaron las reglas de Cohen ^28^.

### Análisis por subgrupos

Para este análisis se consideraron tres variables clave: la intensidad del ejercicio, la supervisión del mismo y la duración total en horas. En relación con la intensidad, se establecieron tres categorías: alta o vigorosa (≥ 6 METs, frecuencia cardíaca máxima ≥ 70%), baja o moderada (< 6 METs, frecuencia cardíaca máxima < 70%) ^14^ y no reportada. Por otro lado, se evaluó si el ejercicio, según el ensayo, fue supervisado por algún profesional de la salud, entrenador personal de manera presencial o virtual. Además, se consideró la duración total del ejercicio en horas, dividiéndose en dos grupos: mayores de 30 horas y menores o iguales a 30 horas al mes, según un estudio por Shiroma *et al.*, que determinaron que aquellos pacientes que realizaban menos de 60 minutos tuvieron un incremento del peso mayor o igual del 3% en los siguientes cinco años en adultos mayores ^29^.

### Evaluación del riesgo de sesgo

Los ensayos clínicos aleatorizados se evaluaron mediante la herramienta Cochrane de riesgo de sesgo para ensayos aleatorios (RoB 2) ^30^. Esta herramienta considera diversos dominios en los que podría haber surgido el sesgo, como el proceso de asignación al azar, desviaciones de las intervenciones planificadas (efecto de la asignación de la intervención), la falta de datos de resultado, la medición de resultados y la selección de resultados informados. Para cada dominio, se utilizó un algoritmo basado en el juicio para determinar si existía bajo riesgo, alguna preocupación o alto riesgo de sesgo. Se consideró que un ensayo clínico aleatorizado tenía alto riesgo de sesgo si presentaba alto riesgo en cualquiera de los dominios evaluados. La evaluación RoB 2 fue realizada de manera independiente por dos autores (MCL, MMT), y las discrepancias se resolvieron mediante discusión o consulta con un tercer autor (IPT).

### Análisis estadístico

Todos los metaanálisis se realizaron utilizando el método de varianza inversa y el modelo de efectos aleatorios. La varianza entre los estudios (τ2) se estimó mediante con la herramienta DerSimonian-Laird. El efecto agrupado de cada resultado se determinó mediante la diferencia de medias estandarizada (DME) de los valores de cambio entre los grupos de intervención y control con los intervalos de confianza del 95% (IC 95%). Cuando no se reportó la desviación estándar de las diferencias de medias, se usó el método del Manual Cochrane a través de la imputación de coeficientes de correlación^31^. La heterogeneidad entre los estudios se evaluó mediante la estadística I2. La heterogeneidad se definió como baja si I2 < 30%, moderada si I2 = 30-60%, y alta si I2 > 60%. Todo lo anterior se presentó como un gráfico *forest plot* para cada resultado.

Para investigar la fuente de heterogeneidad, el análisis de subgrupos se consideró por separado para cada subgrupo y se realizó de acuerdo con la intensidad del ejercicio, la supervisión del rendimiento del ejercicio y el tiempo total de intervención. Se usó la función metacont del paquete estadístico R 4.3.0 (www.r-project.org).

### Certeza de la evidencia

Para evaluar la calidad de la evidencia para todos los resultados, se utilizó el enfoque de Evaluación, Desarrollo y Desarrollo de la Calificación de las Recomendaciones (GRADE) ^32^. Al emplear la metodología GRADE, se tuvieron en cuenta los siguientes criterios para evaluar la calidad de los estudios: riesgo de sesgo, inconsistencia, imprecisión (se tomó en cuenta una diferencia de una DE como diferencia mínima), sesgo de publicación, gran magnitud del efecto, gradiente dosis-respuesta, efecto de la confusión residual plausible y nivel de indirectividad. La tabla resumen de hallazgos (SOF) se generó mediante el software GRADE pro (https://www.gradepro.org). La evaluación de certeza de la evidencia mediante esta escala fue realizada por los autores MMT y MCL, en duplicado y siguiendo las recomendaciones de GRADE.

### Aspectos éticos

Todos los ensayos clínicos incluidos en el estudio tuvieron la aprobación ética necesaria. La información extraída paso por un control de calidad en cada etapa para asegurar la fidelidad de los datos presentados. Adicionalmente se preparó un protocolo antes del inicio de la investigación, el cual se encuentra disponible públicamente en PROSPERO (https://www.crd.york.ac.uk/prospero/) con el código de registro CRD42021250115 y garantiza la transparencia del proceso.

## RESULTADOS

Se identificaron un total de 17 440 resúmenes en las bases de datos y se eliminaron 11 936 duplicados. Después de evaluar por título y resumen, se dejaron 299 para la evaluación a texto completo y seis no fueron accedidos. Finalmente, siete artículos cumplieron con los criterios de inclusión. El proceso de selección se describe en la figura 1.

**Figura 1:** Diagrama de selección de estudios PRISMA.

### Características de los estudios incluidos

Las principales características de los siete estudios se resumen en el material suplementario 3. Se evaluaron un total de 386 participantes con una edad media que osciló entre 62,9 y 73,2 años. Dos estudios procedían de Canadá. En cuanto al tipo de intervención, todos los estudios tuvieron como intervención a las caminatas al aire libre o en caminadora ^33^^-^^39^. La duración de la intervención varió de 12 a 24 semanas y, entre los que informaron la intensidad del ejercicio, el 50% fueron de alta intensidad. De los siete ensayos incluidos, uno demostró un alto riesgo de sesgo en general, como se indica en el material suplementario 4.

### Resultados primarios

Se llevaron a cabo metaanálisis para cada resultado con los siete ensayos incluidos. Los resultados se detallan a continuación: 

En relación con la glucosa en ayunas, en cinco ECA ^34^^,^^35^^,^^40^^-^^44^ que incluyeron 259 participantes, el ejercicio aeróbico redujo significativamente los valores de glucosa (DME: -1,76; IC 95%: -2,78; -0,74; I2 = 91%) en comparación con el grupo control (Figura 2A).

En cuanto a la hemoglobina glicosilada, en cuatro ECA ^34^^,^^37^^,^^45^^-^^49^ que incluyeron a 288 participantes, el ejercicio aeróbico redujo significativamente los valores de HbA1c (DME: -0,63; IC 95%: -0,87; -0,39; I2 = 0%) en comparación con el grupo control (Figura 2B).

**Figura 2:** Diferencia de medias estandarizada entre el ejercicio aeróbico y los grupos de control, un metaanálisis del modelo de efectos aleatorios. (A) Glucosa en sangre en ayunas; (B) Hemoglobina glicosilada.

Con respecto a la presión arterial sistólica, en cuatro ECA, que incluyeron 220 participantes ^33^^,^^34^^,^^36^^,^^39^^,^^48^^,^^50^ el ejercicio aeróbico redujo significativamente los valores de PAS (DME: -1,67; IC 95%: -2,74; -0,61; I2 = 90%) en comparación con el grupo control (Figura 3A).

En cuanto a la presión arterial diastólica, en cuatro ECA ^33^^,^^34^^,^^36^^,^^39^^,^^47^^,^^50^ que incluyeron 220 participantes, el ejercicio aeróbico redujo significativamente los valores de PAD (DME: -1,47; IC 95%: -2,34; -0,60; I2 = 86%) en comparación con el grupo control (Figura 3B).

**Figura 3:** Diferencia de medias estandarizada entre el ejercicio aeróbico y los grupos de control, un metaanálisis del modelo de efectos aleatorios. (A) Presión arterial sistólica; (B) Presión arterial diastólica

Con respecto a los niveles de HDL, en cinco ECA ^35^^-^^39^^,^^47^, que incluyeron 319 participantes, el ejercicio aeróbico aumentó significativamente sus valores (DME: 0,41; IC 95%: 0,11; 0,72; I2 = 41%) en comparación con el grupo control (Figura 4A).

Con respecto a los niveles de triglicéridos, en cinco ECA (35-39,48) que incluyeron 319 participantes, el ejercicio aeróbico redujo significativamente los valores de triglicéridos (DME: -0,34; IC 95%: -0,67; -0,01; I2 = 48%) en comparación con el grupo control (Figura 4B).

**Figura 4:** Diferencia de medias estandarizada entre el ejercicio aeróbico y los grupos de control, un metaanálisis del modelo de efectos aleatorios. (A) Lipoproteína de alta densidad; (B). Triglicéridos

No se realizó un metaanálisis de la circunferencia de la cintura ya que solo se encontró un ECA ^38^; sin embargo, hubo una reducción significativa entre el grupo de ejercicio aeróbico y el grupo de control (p < 0,001).

Con respecto al análisis de sensibilidad, ningún estudio individual afectó significativamente el resultado agregado en ningún resultado (ver material suplementario 5).

### Efectos adversos

Dos de los siete ensayos informaron sobre algunos efectos adversos (material suplementario 3). Madden *et al.*^34^ describieron un caso de síncope relacionado con el masaje carotídeo que ocurrió tanto como después de la realización del ejercicio. Por su parte, Parra-Sanchez *et al.*^36^ reportaron un caso de esguince en el grupo de intervención, y dos casos de accidentes cerebrovasculares, uno en el grupo de intervención y el otro en el grupo de control. Los efectos adversos no se consideraron como resultados adicionales debido a la escasez de los mismos, en los estudios incluidos.

### Análisis por subgrupos

Se realizó un análisis de subgrupos para evaluar los resultados en base a algunas variables como la intensidad del ejercicio, la supervisión del rendimiento del ejercicio y el tiempo total de la intervención (material suplementario 6).

Con respecto a los niveles de glucosa en ayunas, la presión arterial sistólica y diastólica, todos los subgrupos fueron estadísticamente significativos, excepto la mayoría de los ensayos que no informaron la intensidad del ejercicio.

Con respecto a la disminución de los triglicéridos, los ensayos que no informaron la intensidad del ejercicio, aquellos con una duración total de la intervención menor o igual a 30 horas, y aquellos con o sin la supervisión del cumplimiento del ejercicio no tuvieron diferencias significativas entre la intervención y el grupo control.

Acerca de la elevación del HDL, sólo los ensayos que no supervisaron el rendimiento del ejercicio tienen una diferencia estadísticamente significativa entre el grupo de intervención y el grupo de control. En cuanto a la disminución de la HbA1c, todos los subgrupos fueron significativos.

### Certeza de la evidencia

La certeza de evidencia se evaluó en cuatro niveles, desde alta hasta muy baja, según el grado de confianza en la estimación del efecto. Los resultados muestran que la certeza de evidencia es baja para la disminución de glucosa en ayuna y baja para PAS y PAD. Esto debido a la alta heterogeneidad entre los estudios y la diferencia de tipo de ejercicio aeróbico. La certeza de evidencia para el colesterol asociado a lipoproteínas de alta densidad, triglicéridos y HbA1c es muy baja. Esto debido a que los intervalos de confianza para estos efectos son imprecisos, hay preocupación en diferentes dominios de la evaluación del riesgo de sesgo, así como diferencia entre los tipos de ejercicio aeróbico metaanalizados (Tabla 1).

**Tabla 1:** Certeza de evidencia (GRADE).

IC: intervalo de confianza; DE: desviación estándar; DME: diferencia de media estandarizada.

a El riesgo en el grupo intervención (y su intervalo de confianza del 95%) están basados en el riesgo asumido en el grupo control y el efecto relativo de la intervención (y su intervalo de confianza del 95%), ^b^ el valor de l^2^ es 91% en esta variable, ^c^ diferente tipo y duracion de las intervenciones, ^d^ el valor de I2 es 90% en esta variable, ^e^ el valor de I2 es 86% en esta variable, ^f^ intervalos de confianza imprecisos, ^g^ la mayoría de los estudios tienen «algunas preocupaciones» en el dominio uno

Grupos de Certeza de evidencia GRADE. Certeza alta: estamos muy seguros de que el efecto verdadero se aproxima al de la estimación del efecto. Certeza moderada: tenemos una confianza moderada en la estimación del efecto: es probable que el verdadero efecto esté cerca de la estimación del efecto, pero existe la posibilidad de que sea sustancialmente diferente. Certeza baja: nuestra confianza en la estimación del efecto es limitada: el verdadero efecto puede ser sustancialmente diferente de la estimación del efecto. Certeza muy baja: tenemos muy poca confianza en la estimación del efecto: es probable que el verdadero efecto sea sustancialmente diferente de la estimación del efecto.

## DISCUSIÓN

Esta revisión sistemática proporciona una visión completa y actualizada de los efectos del ejercicio aeróbico sobre los componentes del síndrome metabólico en la población geriátrica. Se encontró una disminución significativa en la glucosa en ayunas, la hemoglobina glicosilada, los triglicéridos, la PAS, la PAD y el aumento de los valores de HDL. Además, cuatro de los 386 participantes en el estudio presentaron eventos adversos y en un participante del grupo de intervención, el evento fue grave (accidente cerebrovascular). Adicionalmente, todos los resultados primarios evaluados tuvieron un nivel de certeza ya sea baja o muy baja.

De la síntesis de cinco ECA, se obtuvo que los valores de glucosa disminuyeron con la práctica de ejercicio aeróbico, nuestros hallazgos fueron similares a los reportados por la Kumar *et al*. ^44^ quienes evaluaron 11 ECA con una población adulta de pacientes diabéticos y encontraron una disminución tanto en la glucosa en ayunas como en la HbA1c. Estos resultados pueden explicarse por el hecho de que una rutina de entrenamiento frecuente mejora el rendimiento hepático al reducir la acumulación de lípidos, mejorar la resistencia a la insulina en este órgano y disminuir la producción nocturna de glucosa hepática ^45^, junto con una mayor actividad de las enzimas mitocondriales (mejorando así la energía muscular) ^46^.

Los resultados de la síntesis de cuatro ECA en nuestra revisión, así como otras revisiones sistemáticas con características similares, son consistentes cuando se utiliza la HbA1c como medida del control glucémico. Estos resultados son semejantes a lo encontrado en otras revisiones sistemáticas con características parecidas. Por ejemplo, en una población similar, Pan *et al*. ^38^ demostraron que el ejercicio aeróbico supervisado tuvo un mayor efecto en la HbA1c en comparación con el ejercicio de resistencia o combinado. Además, Hagag *et al*. ^39^ encontraron una diferencia de medias de -0,46 en la HbA1c a favor del ejercicio aeróbico en una muestra de 972 participantes. Se cree que la diferencia en el sustrato utilizado durante cada tipo de ejercicio podría explicar estos resultados. Durante el ejercicio de alta intensidad se utiliza más glucosa, mientras que durante el ejercicio de intensidad moderada se utilizan más ácidos grasos ^41^.

En relación al metabolismo lipídico, nuestra revisión sintetizó el resultado de cinco ECA, los cuales apoyan la relación entre el ejercicio aeróbico y la normalización de los valores de lípidos y lipoproteínas. Específicamente, se observó un aumento de los valores de HDL y una disminución de los triglicéridos después de la intervención. Estos resultados son consistentes con otras revisiones como la de Schwingshacklet *et al*. ^47^, quienes reportaron mejoras en todos los componentes del perfil lipídico después de la práctica de diferentes tipos de ejercicio durante ocho semanas, especialmente con el ejercicio combinado. El aumento de HDL con el ejercicio se debe a la asociación entre el ejercicio aeróbico y el aumento de la actividad de la lipoproteína lipasa, lo que se traduce en un aumento de la lipólisis de lipoproteínas ricas en triglicéridos, que puede ser un paso inicial en los niveles más altos de HDL sérico. Además de lo anterior, hay una reducción en la actividad de la lipasa hepática y la concentración sérica de proteína de transferencia de éster colesterilo (CETP), siendo esta última la que cataliza el flujo de transformación del colesterol HDL al colesterol VLDL y colesterol LDL ^48^.

A partir de la síntesis de cuatro ECA se ha informado que el ejercicio aeróbico disminuye los valores de PAS y PAD. En este sentido, Punia *et al*. ^49^ encontraron que el ejercicio aeróbico tuvo un efecto significativo en la disminución de la PAS y la PAD en adultos de la India. El mecanismo por lo que esto ocurre es una reducción de la resistencia vascular periférica lo que lleva a una disminución de los valores de presión arterial sistólica y diastólica después del entrenamiento ^17^. Sin embargo, según otro estudio, los mecanismos por los cuales el ejercicio reduce la presión arterial todavía no se comprenden completamente. Se han propuesto diversas vías posibles, incluyendo la reducción de la inflamación, la disminución del daño oxidativo, el control de la sensibilidad al sodio y la disminución de la rigidez arterial ^46^.

En relación a los efectos adversos, se registró un evento adverso grave (accidente cerebrovascular) en uno de los 386 participantes incluidos en el estudio. En otro ECA que evaluó diferentes rutinas de entrenamiento en 221 individuos, Church *et al*. ^50^ reportaron cinco eventos cardiovasculares, pero ninguno relacionado con el ejercicio físico. Sin embargo, se debe tener en cuenta que en estudios previos se han reportado mayores efectos adversos en adultos mayores con diabetes, posiblemente debido a su fragilidad y mayor prevalencia de comorbilidades ^50^. Por lo tanto, se debe considerar con precaución los resultados de los ensayos, especialmente teniendo en cuenta que la edad media de los participantes en los mismos oscilo entre los 60 y 70 años.

Teniendo en cuenta que la diabetes es uno de los principales problemas de salud pública ^10^, nuestro estudio muestra que el ejercicio aeróbico, una medida de bajo costo y relativamente fácil de implementar, ayuda en gran medida a mejorar los componentes del síndrome metabólico en la población mayor. Su implementación en el sistema sanitario como un programa dedicado a la actividad aeróbica podría potencialmente reducir significativamente la aparición de complicaciones y evitar el avance a corto plazo a eventos cardiovasculares, los cuales tienen una alta mortalidad y un alto riesgo de secuelas irreversibles y dependencia. Además, se pueden agregar los efectos beneficiosos indirectos que reducen en gran medida la fragilidad de este grupo de edad. Sin embargo, debido a que los estudios analizados se basan en *outcomes* subrogados, no se puede confirmar la relevancia clínica. Por último, y no menos importante, probablemente ayudaría a reducir la dosis de fármacos hipoglucemiantes e hipotensores, lo que podría traducirse en un menor costo en el mantenimiento de las enfermedades crónicas. Nuestra revisión es relevante porque, hasta donde sabemos, es la primera que analiza los efectos del ejercicio aeróbico sobre los componentes del síndrome metabólico en la población anteriormente mencionada. Esto ayudaría a orientar el manejo futuro e implementar este tipo de ejercicio en la práctica diaria de estos pacientes.

Nuestra investigación tuvo algunas limitaciones. En primer lugar, hubo un alto porcentaje de estudios con riesgo alto o moderado de sesgo. En segundo lugar, las características de las intervenciones fueron heterogéneas, sin embargo, se realizó un análisis de subgrupos según el tiempo de intervención, la supervisión y la intensidad, que la mayoría no mostró diferencias, estos resultados deben considerarse con precaución debido al bajo número de ensayos. En tercer lugar, este estudio abarcó siete ECA, la gran mayoría de estos provenía de países asiáticos y tuvieron un número relativamente bajo de participantes, aunque se realizó un análisis sensible excluyendo cada estudio, lo cual no mostró diferencias significativas. Sin embargo, más estudios de población europea y americana son necesarios. En cuarto lugar, una de las limitaciones fue la heterogeneidad de los estudios incluidos en el metaanálisis tal como la glucosa en sangre en ayunas y presión arterial sistólica y diastólica.

Finalmente, no fue posible evaluar el efecto específico de diferentes rutinas de ejercicio aeróbico porque la mayoría de los ECA incluidos, la intervención fue la misma o parecida (cinta de correr, caminar, uso de caminadora y bicicleta ergométrica). No obstante, todos estos tipos de ejercicios producen estímulos en largas porciones de músculo esquelético, logrando con esto, provocar cambios favorables en el sistema cardiovascular ^24^.

En conclusión, los estudios clínicos disponibles muestran una disminución significativa en los valores de glucosa en ayunas, triglicéridos, hemoglobina glicosilada, PAS y PAD, así como una pequeña modificación de los triglicéridos y HDL en adultos mayores con DM2 que realizaron ejercicio aeróbico en comparación con los controles sin ejercicio, por lo que sugerimos esta intervención en esta población teniendo en cuenta el nivel de certeza de evidencia siendo baja para la glucosa en ayunas, la presión arterial sistólica y diastólica, y muy bajo para los demás resultados. además de pocos efectos adversos. Sin embargo, estos resultados deben ser interpretados con cautela debido al uso de marcadores subrogados. Por último, se recomienda realizar ensayos clínicos de mejor calidad metodológica, multicéntricos y con muestras más grandes para que pueda dar una correcta representación de los efectos del ejercicio aeróbico.
